# MOF/Polymer-Integrated Multi-Hotspot Mid-Infrared Nanoantennas for Sensitive Detection of CO_2_ Gas

**DOI:** 10.1007/s40820-022-00950-1

**Published:** 2022-10-22

**Authors:** Hong Zhou, Zhihao Ren, Cheng Xu, Liangge Xu, Chengkuo Lee

**Affiliations:** 1 Department of Electrical and Computer Engineering, National University of Singapore, Singapore, 117583 Singapore; 2 Center for Intelligent Sensors and MEMS (CISM), National University of Singapore, Singapore, 117608 Singapore; 3 NUS Suzhou Research Institute (NUSRI), Suzhou, 215123 People’s Republic of China

**Keywords:** Metal–organic framework, Gas detection, Mid-infrared nanoantennas, Multi-hotspot, Loss engineering

## Abstract

Metal–organic frameworks (MOFs) have been extensively used for gas sorption, storage and separation owing to ultrahigh porosity, exceptional thermal stability, and wide structural diversity. However, when it comes to ultra-low concentration gas detection, technical bottlenecks of MOFs appear due to the poor adsorption capacity at ppm-/ppb-level concentration and the limited sensitivity for signal transduction. Here, we present hybrid MOF-polymer physi-chemisorption mechanisms integrated with infrared (IR) nanoantennas for highly selective and ultrasensitive CO_2_ detection. To improve the adsorption capacity for trace amounts of gas molecules, MOFs are decorated with amino groups to introduce the chemisorption while maintaining the structural integrity for physisorption. Additionally, leveraging all major optimization methods, a multi-hotspot strategy is proposed to improve the sensitivity of nanoantennas by enhancing the near field and engineering the radiative and absorptive loss. As a benefit, we demonstrate the competitive advantages of our strategy against the state-of-the-art miniaturized IR CO_2_ sensors, including low detection limit, high sensitivity (0.18%/ppm), excellent reversibility (variation within 2%), and high selectivity (against C_2_H_5_OH, CH_3_OH, N_2_). This work provides valuable insights into the integration of advanced porous materials and nanophotonic devices, which can be further adopted in ultra-low concentration gas monitoring in industry and environmental applications.

**Supplementary Information:**

The online version contains supplementary material available at 10.1007/s40820-022-00950-1.

## Highlights

- A loss-engineered multi-hotspot strategy is proposed by integrating all major optimization methods to improve the sensitivity and signal intensity of plasmonic nanoantennas for mid-infrared (MIR) absorption-based molecule sensing.
- The physi-chemisorption mechanism of hybrid metal–organic framework (MOF)-polymers is demonstrated to break through the limit of detection for MIR gas sensing.
- MOFs and nanoantennas are successfully integrated to achieve high-performance gas detection, including low detection limit, high sensitivity (0.18%/ppm), excellent reversibility (variation within 2%), high selectivity, and nm-level optical interaction length.

**Supplementary Information:**

The online version contains supplementary material available at 10.1007/s40820-022-00950-1.

## Graphical abstract

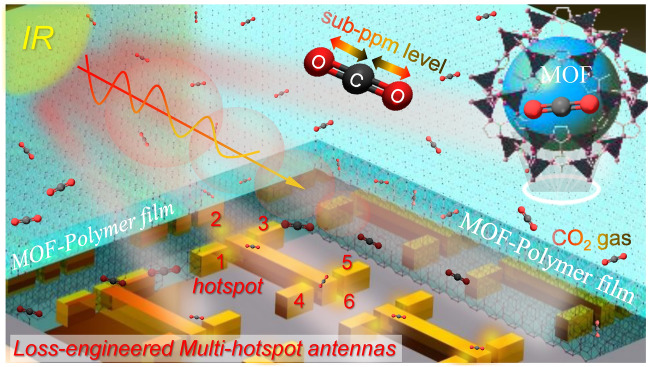

## Introduction

Metal–organic frameworks (MOFs) are extended crystal structures composed of metal cations linked by multitopic organic ions or molecules [1–3]. Due to the diversity and connection network of metal ions, organic linkers, and structural motifs, MOFs are unparalleled in terms of internal surface area, tenability, structural diversity as well as the range of chemical and physical properties [4–7]. Thanks to these advantages, MOFs have been widely used in a variety of applications including gas storage and separation [8, 9], proton conduction [10], drug delivery [11, 12], and sensing [13, 14]. Among them, the exploration of MOFs for sensing applications is challenging and involves multidisciplinary technology on account of the requirement for signal transduction to detect changes within MOFs [15]. Despite challenges, the exceptional properties of MOFs such as large surface area, extensive porosity, and high crystallinity should constitute important advantages over other candidate classes of gas sensing materials [16]. Thanks to the great efforts invested in the field of MOFs, breakthroughs have been made in signal transduction technologies, including optical (photoluminescence [17], localized surface plasmon resonance [18], solvochromicity [19], etc.) and mechanical solutions (quartz crystal microbalances [20], and microcantilever schemes [21], etc.). However, signal transduction remains a major challenge for the sensing implementation of MOFs owing to the diversity of detection requirements; especially, ultrasensitive signal transduction protocols are highly desirable for the advancement of MOFs and gas detection in the field of medical diagnostics, food quality control, occupational safety, and environmental monitoring.

Nanoantennas, the artificial sub-wavelength structures with highly engineered optical properties [22–30], can excite strong near fields with thousands-fold intensity enhancement by locally enhancing the manipulation of light at subwavelength scales [31–40]. The strong near field is sensitive to subtle changes in nearby substances via plasmon–molecule interaction [41–47], making it a competitive candidate for ultrasensitive signal transduction of MOFs [48]. According to recent reports [49–52], the implementation of nanoantenna-based signal transduction of MOFs for gas detection faces two technical challenges. One is the small variation in MOFs to the target gas, that is, the poor gas adsorption capacity of MOFs and the low infrared (IR) absorption of MOF molecules. The other is the relatively low detected IR intensity of MOFs in the near-field region of the nanoantenna; hence, it results in poor plasmon–molecule interactions. In terms of the absorbed amount of target gas molecules in MOFs, it mainly relies on the type of uptake gases (e.g., carbon dioxide, CO_2_) by physisorption of MOFs through Lewis acid open metal sites [53] or molecular size confinement [54]. This physisorption-induced variation in MOFs is small because of the low gas adsorption at low concentrations, and it is difficult to detect by the nanoantenna sensing technology. The currently reported detection limit based on this technique is 80 ppm [49], which is not good enough for certain industrial applications. Therefore, considering the dramatic change of absorbent structure by chemisorption, simple methods to expand the chemisorption function of MOFs to amplify the variation in gas adsorption and IR absorption in MOFs are the promising direction for further investigation.

Currently, there are three methods of enhancing the plasmon–molecule interactions in the nanoantenna sensing technology. These methods include increasing the spatial overlapping of molecules to the near field [55–58], enhancing near-field intensity [59–62], and loss optimization (Note S1) [58, 63, 64]. The introduction of a MOF coating layer on top of nanoantenna as an enrichment material to concentrate gas molecules in the near field is a new spatial overlapping method to increase the interaction between gas molecules and infrared radiation in the near-field region. The widely used approach to increase the near-field intensity is the proximity of adjacent nanoantennas to form nanogaps, where strong near-field interaction occurs between the adjacent nanoantennas owing to their capacitive coupling [65–67]. Loss engineering is the second method to enhance the interactions by folding the nanoantenna structure to tune the loss of the plasmon–molecule interactions system [63]. It has received increasing attention owing to its advantage of improving performance without extraordinary fabrication cost, e.g., the fabrication process of making a few tens of nanometer gaps created among nanorods. In general, the above methods effectively improved the plasmon–molecule interactions when they are demonstrated independently rather than in combination with each other [68]. However, it is quite challenging to maximally strengthen the interaction by simultaneously adopting the above methods due to the contradiction of these methods caused by different optimization principles. More specifically, regarding the near-field optimization of constructing nanogaps, the adjacent nanoantennas parallel to the polarization of incident light are in a superradiant “bright” mode which can couple efficiently to the incident optical field [69]. The coupling efficiency of bright mode antennas is diminished by the increased losses in antennas due to the dissipation of the radiated field [70]. However, loss increment in loss optimization is the key to enhancing the plasmon–molecule interaction. Therefore, the improvement achieved by near-field optimization could be attenuated by loss optimization because of the loss increment. To date, although the integration of spatial overlapping optimization with near-field optimization or loss optimization has been investigated [58, 71], the combination of near-field optimization and loss optimization methods has not been experimentally exploited because of the near-field weakening caused by loss increment (Fig. S1). Therefore, to achieve ultrasensitive signal transduction of MOFs, it is difficult but necessary to push the limits of plasmon–molecule interaction improvement further by reconciling the contradiction between near-field enhancement and loss optimization.

In this work, we present a paradigm integrating physi-chemisorption of MOF hybrids with nanoantenna-based ultrasensitive signal transduction for sensitive CO_2_ gas detection. In terms of MOFs, amino groups are introduced into MOFs through post-synthetic modification to expand the chemisorption function of the MOFs while maintaining their structural integrity for physisorption, thereby greatly amplifying the variation in gas adsorption and IR absorption in MOFs to gases. In terms of ultrasensitive signal transduction of MOFs, a nanogap-coupled multi-hotspot nanoantenna strategy is proposed to reconcile the contradiction between near-field enhancement and loss optimization. More specifically, subradiant “dark” mode nanoantennas, which interact weakly with the incident optical field, are coupled to bright mode nanoantennas through well-engineered nanogaps that are located beside the “lightning-rod effect” region (hotspot) of nanoantennas. The nanogap is designed beside the hotspot of bright mode antennas to excite multiple new high-intensity hotspots. Utilizing the increase in hotspot number to compensate for the intensity weakening of near field caused by loss optimization, we achieve near-field optimization and loss optimization simultaneously. According to the best of our knowledge, it is the first time to achieve the integration of all major optimization methods in plasmonic nanoantennas (Table S1). Benefiting from pioneering efforts in MOFs modification and nanoantenna-based ultrasensitive signal transduction, we demonstrate the competitive advantages of our strategy in state-of-the-art CO_2_ gas sensors, including 1 ppm detection limits, high sensitivity, and nm-level optical interaction length for miniaturization.

## Experimental

### Numerical Simulations

The spectral and near-field simulations were implemented by using a finite-difference time-domain (FDTD)-based commercial software package (FDTD Solutions, Lumerical Inc). In the simulation setting, the incident radiation source used a plane wave light source whose polarization was set along the length of the bright mode nanoantennas. Periodic boundary condition was chosen to model the periodicity in the x- and y-directions of nanoantennas, and the boundary condition in the z-direction was set to a perfectly matched layer. The refractive index of CaF_2_ was set to 1.38, and that of Au was taken from Palik et al. The complex refractive index of PMMA was calculated according to its measured IR absorption spectrum by using $\varepsilon (\omega ) = \varepsilon_{\infty } + \sum\nolimits_{i}^{n} {\frac{{S_{i} }}{{\omega_{0}^{2} - \omega^{2} - j\omega \gamma }}}$, where *ε*_∞_ is the high-frequency constant term, *S*_*i*_ the oscillator strength, *ω*_0_ the oscillator resonance frequency, and *γ* the damping frequency. The thin titanium (Ti) adhesion layer was omitted from the simulation, and we simulate near-field penetration depth using a 3D frequency domain power monitor.

### Device Nanofabrication and MOF Modification

The nanofabrication began with the ultrasonic cleaning of a CaF_2_ wafer for 10 min in acetone. Then, it was rinsed through isopropanol (IPA) and dried with nitrogen, followed by the oxygen plasma treatment for 5 min. After cleaning, the CaF_2_ wafer was spin-coated with a ~ 200-nm-thick layer of PMMA e-beam lithography resist. After thermal bakes, a commercial electron-conducting polymer (Espacer 300Z from Showa Denko Singapore) was spin-coated at a speed of 2000 rpm to eliminate charge accumulation during e-beam exposure. Then, the nanoantenna pattern was exposed using the e-beam lithography technique. After exposure, the sample was sequentially immersed in deionized water, MIBK/IPA (1:3) mixture, and IPA to complete the development of the resist. After development, Ti (5 nm) and Au (70 nm) were sequentially deposited on the surface of the developed sample using e-beam evaporation. Finally, according to the lift-off process, the sample was immersed in acetone for 24 h to remove the unexposed resist and obtain nanoantenna patterns. In terms of the MOF modification, it began with the preparation of zeolitic imidazolate framework (ZIF-8) solutions in methanol. Then, it was sonicated for 10 min at 50% energy in an ultrasonic cleaner to obtain fine crystals. After that, low molecular weight branched polyethyleneimine (PEI) was added to the MOF solution by using a pipette, followed by stirring with a magnetic stirrer for 4 h to allow the amino group and MOF to interact sufficiently. Then, the modified MOF hybrids were spin-coated onto the device surface to complete the preparation of our MOF-PEI-functionalized multi-hotspot platform.

### Optical Measurement

The measurement of the IR spectrum of our platform was performed on a Fourier transform infrared spectrometer coupled to an infrared microscope. The microscope was equipped with a liquid-nitrogen-cooled mercury cadmium telluride detector. The resolution of the test was set to 4 cm^−1^, and the number of scans per measurement was 20. The measurement area of the antenna array was set to 100 × 100 μm^2^ by controlling knife-edge apertures. The background in reflection mode was collected on a gold mirror, and the background in transmission mode was collected on a blank CaF_2_ substrate. Nitrogen gas is continuously introduced into the microscope through designed accessories to ensure that the optical path in the instrument is protected by nitrogen gas.

### Gas Detection Setup and Data Processing

The gas detection setup was shown in Note S2. First, the target CO_2_ gas or N_2_ gas enters the mixing chamber through gas mass flow controllers (MFC), which were adjusted in real time to obtain the desired gas flow rates. Then, the gas flowing out of the MFC is quickly mixed uniformly by a fan in the mixing chamber. To obtain gas concentration and humidity information in the mixing chamber, the expensive commercial CO_2_ gas sensor and humidity sensors were employed. After mixing, the gas flowed into the gas cell where our platform was located. The gas cell consists of upper and lower IR barium fluoride windows, a sample holder, and a heating stage, where the sample holder and heating stage are integrated to efficiently heat our sensing platform. In reflection mode, the detector collects IR light that enters and exits from the upper IR window, while in transmission mode, the detector collects IR light that exits from the lower IR window. For the thermal recovery of our sensing platform, the gas cell was heated up to 90 °C and kept for 1.5 min with a continuous N_2_ gas flow. Then, the heating stage was turned off while maintaining N_2_ gas flow and waiting for the cell to cool naturally to room temperature. In terms of data processing, the reference spectrum was set as the spectrum without CO_2_ adsorption. The differential reflection was extracted from the reflection spectrum by subtracting the reference spectrum, which can be written as *D* = ǀ*R*-*R*_reference_ǀ. The total molecular signal of platforms was calculated by *R*_T_ = ∫*λ*_*1*_ *λ*_*2*_*D∙dλ*, where *λ*_*1*_ and *λ*_*2*_ are the beginning and end wavelength of the MOF changing region. The linearity was calculated by *δ*_L_ = Δ*e*/FS × 100%, where Δ*e* is the maximum error in the full scale FS. The enhanced molecular signal is extracted by fitting the baseline of platform spectra using a least squares method.

### Materials and Apparatus

The branched PEI (average M_w_ ~ 800) was purchased from Sigma-Aldrich (Singapore). The ZIF-8 was purchased from ACS Material, LLC. SEM and EDX analysis was performed by using a field emission scanning electron microscope (Hitachi Regulus 8230). The thickness analysis was performed by using an Alpha step (Alpha step-D100, Tencor Inc., Japan). The IR spectrum of PEI film was measured by an FTIR spectrometer (Cary 660, Agilent Technologies) with an infrared microscope (Cary 610, Agilent Technologies). The pattern of nanoantennas was fabricated by using electron beam lithography (Raith GmbH). The metal deposition was performed by using a UHV electron beam evaporation (ATC-T Series, AJA Int.). XRD was analyzed by a Ragiku Smartlab Diffractometer. BET surface area and N_2_ adsorption isotherms were measured by using a volumetric adsorption analyzer (Micromeritics ASAP 2020, USA).

## Results and Discussion

### Design Concept

**Fig. 1:**
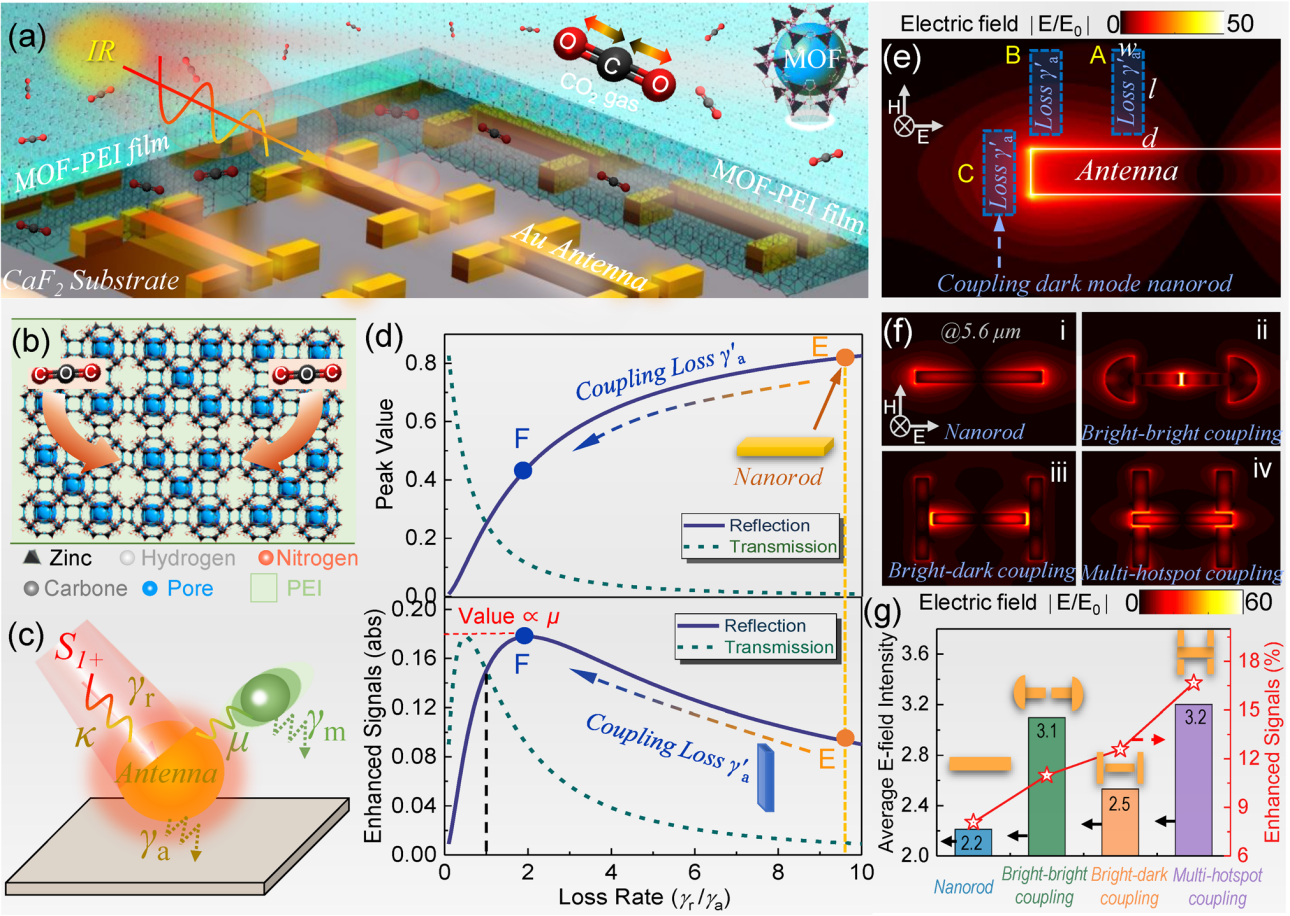
Design concept of the hybrid platform. **a** Schematic representation of the platform consisting of porous MOF-PEI hybrid film, CaF_2_ substrate, and multi-hotspot nanoantennas. MOF-PEI hybrid film functions as gas-selective-trapping materials to adsorb CO_2_ gases both physically and chemically. Nanoantennas provide a strongly enhanced near field for the detection of CO_2_ captured in the hybrid film. **b** Schematic showing the capture of CO_2_ gas molecules by the porous MOF-PEI hybrid film. **c** Coupled mode model of nanoantennas. The bright mode of the nanoantennas is driven by external radiation *S*_*in*_ via coupling rate *κ* and experiences damping at a rate *γ*_*r*_ and *γ*_*a*_. The molecular damping *γ*_*m*_ is coupled to the nanoantennas via a coupling strength *μ*. **d** Optimization of enhanced vibrational signals through loss engineering. Upper panel: spectral peak value versus loss rate of antennas. Lower panel: enhanced vibrational signals versus loss rate of antennas. Nanorod-shaped antennas are at low levels of signal enhancement, and its signal enhancement can be improved by coupling appropriate loss. **e** Different coupling of dark mode loss to bright mode antennas. **f** Electric field distribution of (i) nanorods and nanoantennas in (ii) bright–bright or bright–dark mode coupling (iii: dual-hotspot; iv: multi-hotspot). **g** The average near-field intensity and enhanced molecular signal strength for various configurations described above. Obviously, the average near-field intensity of the bright–dark mode coupling with a dual-hotspot (orange) is lower than that of the bright–bright mode coupling with a single hotspot (green), which is caused by the contradiction between loss and near-field optimization. Our multi-hotspot strategy (purple) can complement the weakened near field and achieve the highest near-field intensity

The platform integrating MOFs and nanoantennas for gas detection, illustrated in Fig. 1a, consists of nanoantennas, calcium fluoride (CaF_2_) substrate, and MOF hybrids. The MOF hybrids function as gas-selective-trapping materials to concentrate and adsorb CO_2_ gases. The variation in MOF hybrids caused by the adsorption of CO_2_ gases is detected by nanoantennas through near-field coupling. More specifically, the IR vibration of MOF hybrids is coupled with the resonance of nanoantennas via near field. When CO_2_ gases are adsorbed by the MOF hybrids, the IR vibration of MOF hybrids changes accordingly, which is reflected in the IR spectrum of the nanoantenna via near-field coupling. Therefore, we can determine the gas information according to the change in the IR spectrum of nanoantennas. Throughout the detection process, the nanoantenna serves as signal transduction to convert the gas changes in MOF hybrids into easily detectable spectral changes. The MOFs we used here are ZIF-8, which is composed of Zn^2+^ atoms linked to imidazolate anions through nitrogen and forms tetrahedral coordination [72]. The aperture between the connecting nets is 3.4 Å, which matches well with the kinetic diameter of CO_2_ molecules, making porous ZIF-8 a good CO_2_ gas adsorption material. However, the adsorption is physical, and it resulted in small variation at low gas concentrations. To maximize the gas-induced changes in MOFs, amino groups in PEI are introduced into MOFs through post-synthetic modification to expand the chemisorption function of the MOFs while maintaining their structural integrity for physisorption (Fig. 1b). The basic amine groups of PEI polymers can chemically react with hard-acidic CO_2_ molecules due to the zwitterion mechanism. The reaction process is reversible since the formed species caused by the CO_2_ absorption can be recovered by heating with negligible PEI loss (Note S3). Additionally, PEI-modified MOFs are easily integrated with nanoantennas through the spin coating process. Notably, the content of PEI polymer in MOF hybrids has a great influence on gas detection. Insufficient PEI polymer could lead to low gas adsorption owing to the lack of reactive adsorption sites, and excessive PEI polymer could prevent the diffusion of CO_2_ molecules into the inner layer of MOF hybrids because of the complete pore filling. Therefore, there is a balance between the physisorption of MOFs and the chemisorption of PEIs, which will be discussed in detail in a later section.

As for the signal transduction using nanoantennas, it is necessary to maximally strengthen the plasmon–molecule interaction by simultaneously adopting all major optimization methods. The main difficulty of this task lies in reconciling the contradiction between near-field optimization and loss optimization. For this task, an analysis of the plasmon–molecular coupling behavior by temporal coupled mode theory (TCMT) is conducted [73]. The TCMT model consists of a single cavity representing the bright mode of the nanoantennas coupled (*κ*) with two ports including incident light (*S*_*1*+_), transmitted light, and reflected light (Fig. 1c). It experiences damping at a rate *γ*_*r*_ and *γ*_*a*_, and the molecular damping *γ*_*m*_ is coupled to the nanoantennas via a coupling strength *μ*. A series of equations describing the plasmon–molecule coupling behavior can be obtained accordingly. With further simplification, the spectral transmission and reflection of the system are obtained. (Note S4)

1 $$T(\omega ) = \left| {\frac{{j(\omega - \omega_{0} ) + \gamma_{a} + \frac{{\mu^{2} }}{{j(\omega - \omega_{m} ) + \gamma_{m} }}}}{{j(\omega - \omega_{0} ) + (\gamma_{a} + \gamma_{r} ) + \frac{{\mu^{2} }}{{j(\omega - \omega_{m} ) + \gamma_{m} }}}}} \right|^{2}$$

2 $$R(\omega ) = \left| {\frac{{\gamma_{r} }}{{j(\omega - \omega_{0} ) + (\gamma_{a} + \gamma_{r} ) + \frac{{\mu^{2} }}{{j(\omega - \omega_{m} ) + \gamma_{m} }}}}} \right|^{2}$$

where *ω*_0_ and *ω*_*m*_ represent the resonant angular frequency of nanoantennas and molecular vibration, respectively. Intensity changes in transmission (*ΔT*) or reflection (*ΔR*) spectra are often utilized to reflect the amount of molecules in vibrational spectroscopy and various sensing applications. Through calculating the differential spectra of the nanoantennas with/without coupled molecules at resonance without detuning, the *ΔT*_*m*_ and *ΔR*_*m*_ intensity can be expressed by (see Note S4 for detailed equation derivation)

3 $$\Delta T_{m} = T(\omega = \omega_{0} ) - T(\omega = \omega_{0} ,\mu = 0) = \frac{{2\mu^{2} }}{{\gamma_{a} \gamma_{m} }}\frac{{\gamma_{r} /\gamma_{a} }}{{(1 + \gamma_{r} /\gamma_{a} )^{3} }}$$

4 $$\Delta R_{m} = \left| {R(\omega = \omega_{0} ) - R(\omega = \omega_{0} ,\mu = 0)} \right| = \frac{{2\mu^{2} }}{{\gamma_{a} \gamma_{m} }}\frac{{(\gamma_{r} /\gamma_{a} )^{2} }}{{(1 + \gamma_{r} /\gamma_{a} )^{3} }}$$

As observed, *T(ω)*, *R(ω)*, Δ*T*, and Δ*T* are all mainly determined by the loss rate *γ*_*r*_/*γ*_*a*_ and coupling strength *μ* between molecules and hotspots. By plotting Eqs. 3 and 4 in Fig. 1d, we can observe that as the loss rate increases, the changes in transmission and reflection are monotonic, while the enhanced signal intensity undergoes an evolution that first increases and then decreases. The trend of the intensity change of the enhanced signal is determined by the loss rate *γ*_*r*_/*γ*_*a*_. Meanwhile, according to Eqs. 3 and 4, the overall amplitude of the enhanced signal is positively related to the coupling strength *μ*. Therefore, to obtain a highly enhanced signal, both the adjustment of *γ*_*r*_/*γ*_*a*_ and the increase in *μ* should be considered, which also indicates the necessity to integrate multiple optimization methods.

In terms of the adjustment of *γ*_*r*_/*γ*_*a*_, conventional methods include changing the bending degree of nanoantennas [66] and adding a metal reflection layer closing to nanoantennas [60]. We propose to use the dark mode nanorod as a loss and couple it to a bright mode nanoantenna through well-engineered nanogaps. By coupling appropriate losses, the antenna undergoes a transition from a low signal enhancement state at the E point to a high signal enhancement state at the F point (Fig. 1d). Meanwhile, by comparing the upper and lower panels of Fig. 1d, it can be found that devices with high reflection/transmission may exhibit low performance in the spectroscopic sensing applications. It indicates that the spectral intensity of the system is not positively correlated with the enhanced molecular signal intensity, i.e., an indicator of plasmon–molecule interaction. Due to the uneven lightning-rod-like distribution of the hotspots of the antenna, the coupling efficiency of the bright and dark modes is distinct at different positions. Therefore, the optimization of nanogap position is crucial. Figure 1e shows several representative positions of the nanogap, including the position away from the antenna terminal (marked as A), the position at the end of the antenna (marked as B), and the position symmetrical to the antenna horizontally (marked as C). When the nanogap moves from position A to position C through position B, the average field intensity of the antenna representing the mode coupling efficiency first increases, then decreases, and finally boosts to the maximum (Note S5). Since the coupling between lossy and bright antennas is achieved through the near field in the nanogap, high average field intensity in nanogap means excellent coupling efficiency. Therefore, position C is the optimal position because of its high coupling efficiency from the perspective of the loss optimization method. However, from the perspective of the near-field optimization method, the configuration at position C is not optimal because the near field is weakened due to the lossy coupling when compared with bright–bright mode coupling. This contradictory situation is caused by different optimization principles of the two methods.

More specifically, the widely used scheme in near-field optimization is to utilize the proximity of adjacent bright mode antennas (bright–bright mode coupling) to excite strong near-field interaction (Fig. 1f-ii), where the addition of an expansion structure at the end of the antenna can further boost the near-field intensity. As a result, a significant improvement in molecular signal enhancement is observed, as shown in the red curve of Fig. 1g. When we use common loss-optimization-based bright–dark mode coupling to improve the antenna performance, a higher molecular signal enhancement is achieved compared to near-field optimization. However, we observe that the near-field intensity of antennas using this loss optimization is greatly attenuated (orange bar of Fig. 1g). Apparently, it is caused by the above-mentioned contradiction between near-field and loss optimization. Although the introduction of loss reduces the near-field strength, the loss-optimization-based nanoantenna still achieves a higher molecular signal enhancement than the nanoantenna using near-field optimization. It reflects that loss optimization has a more significant effect than near-field optimization. Ultra-high molecular signal enhancement can be expected if the attenuation in near-field intensity caused by lossy coupling can be decreased or even eliminated. Here we design the nanogap beside the hotspot of bright mode antennas to excite new high-intensity hotspots (Fig. 1f-iv). By increasing the hotspot number to compensate for the intensity weakening of the near field caused by lossy coupling, we obtain a higher average field intensity than bright–bright mode coupling while implementing loss optimization. It means the contradiction between the near-filed and loss optimization is well reconciled by our multi-hotspot strategy, and as a result, ultra-high molecular signal enhancement is achieved for signal transduction of MOF-PEI hybrids (red curve of Fig. 1g).

### Optimization of Nanoantenna-based Signal Transduction

**Fig. 2:**
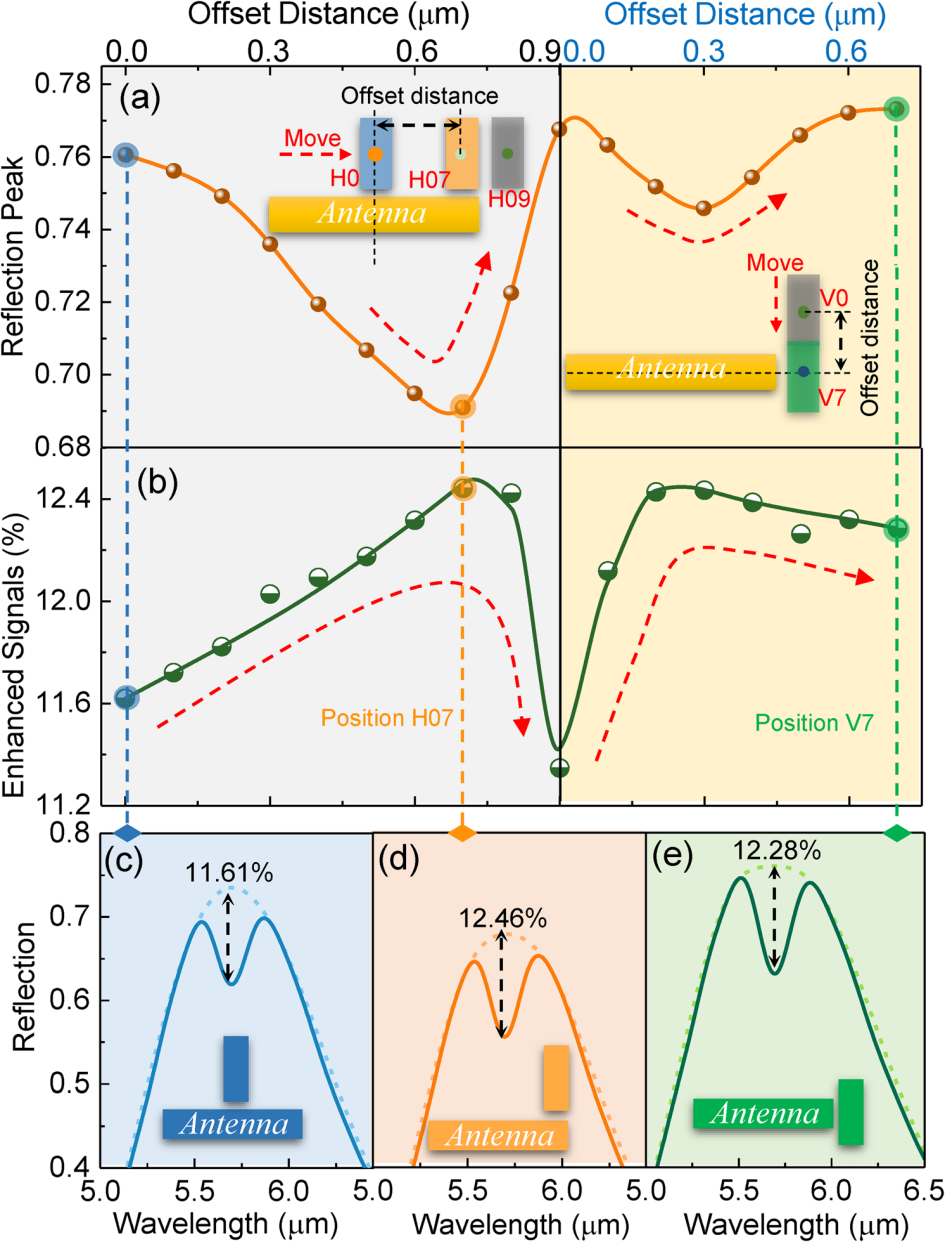
Position optimization of nanogap in bright–dark mode coupling. **a** Simulated spectral reflection peak and **b** enhanced signal profile as the nanogap shifts from the horizontal center H0 to the vertical center V07, where “H” and “V” represent a horizontal or vertical movement, respectively, and the value is the distance moved. Left panel: the nanogap shifts horizontally from H0 to H09; right panel: the nanogap shifts vertically from V0 to V07. The simulated reflection spectra when the dark mode rod is located at **c** the vertical center of the antenna, **d** the right end of the antenna, **e** and the vertical center of the antenna. Dotted line: the baselines of spectra

The optimization of signal transduction is the improvement in molecular signal enhancement of the nanoantenna system, mainly including two basic tasks, namely the position optimization of the nanogap and the dimension optimization of the dark mode antenna. The nanogap position is crucial because it influences the mode coupling efficiency. When the nanogap shifts horizontally from the horizontal center H0 to the end H09 of the bright mode antenna (Fig. 2a), the resonant reflection first decreases and then increases. Correspondingly, the enhanced molecular signal undergoes the opposite evolution (Fig. 2b), where polymethyl methacrylate (PMMA) is selected as the analyte on the basis of its obvious C = O stretching vibration near 5.8 μm (Note S6). According to their relationship in Fig. 1d, this opposite trend indicates that their losses are in the range of points E and F. Similarly, when the nanogap moves vertically from V0 (H09) to the vertical center V07, the resonant reflection and the enhanced signal repeat the same trend. From the perspective of the near field in nanogap (Note S5), the maximum near field during the horizontal shift is formed at the end of antennas, and that during the vertical shift is at the vertical center V07. This trend is consistent with the lightning rod-like distribution of the antenna hotspots. It indicates that nanogaps located at the end-aligned position (Position H07 in Fig. 2a) and vertical center (Position V07) of the bright mode antenna are the potential configurations for high mode coupling efficiency. However, the nanogap located at the vertical center V07 can only enhance the field intensity of the two existing hotspots at both ends of the antenna. In contrast, the nanogap located at H07 can establish four new hotspots beside the two hotspots at the ends of the antenna (Note S7). The newly established hotspots can compensate for the reduced average near-field strength in the antenna system due to the increased losses of loss optimization. Therefore, the antenna configuration with four nanogaps located at H07 is proposed in this work to reconcile the contradiction between optimization methods. Notably, position V2 shows a high signal enhancement comparable to H07 at the horizontal end and V7 at the vertical center. However, similar to V7 at the vertical center, position V2 can only enhance the field intensity of the two existing hotspots at both ends of the antenna. In addition, during the movement of the nanogap, the resonant wavelength and loss of the plasmonic antenna system also shift by introducing different losses (Note S8 and S9). Its changing trend is the same as that of the reflection peak. Therefore, to ensure the non-detuning between the molecular vibration and the antenna resonance (Fig. 2c-e), the length of the bright mode antenna is fine-tuned in the investigation of Fig. 2.

**Fig. 3:**
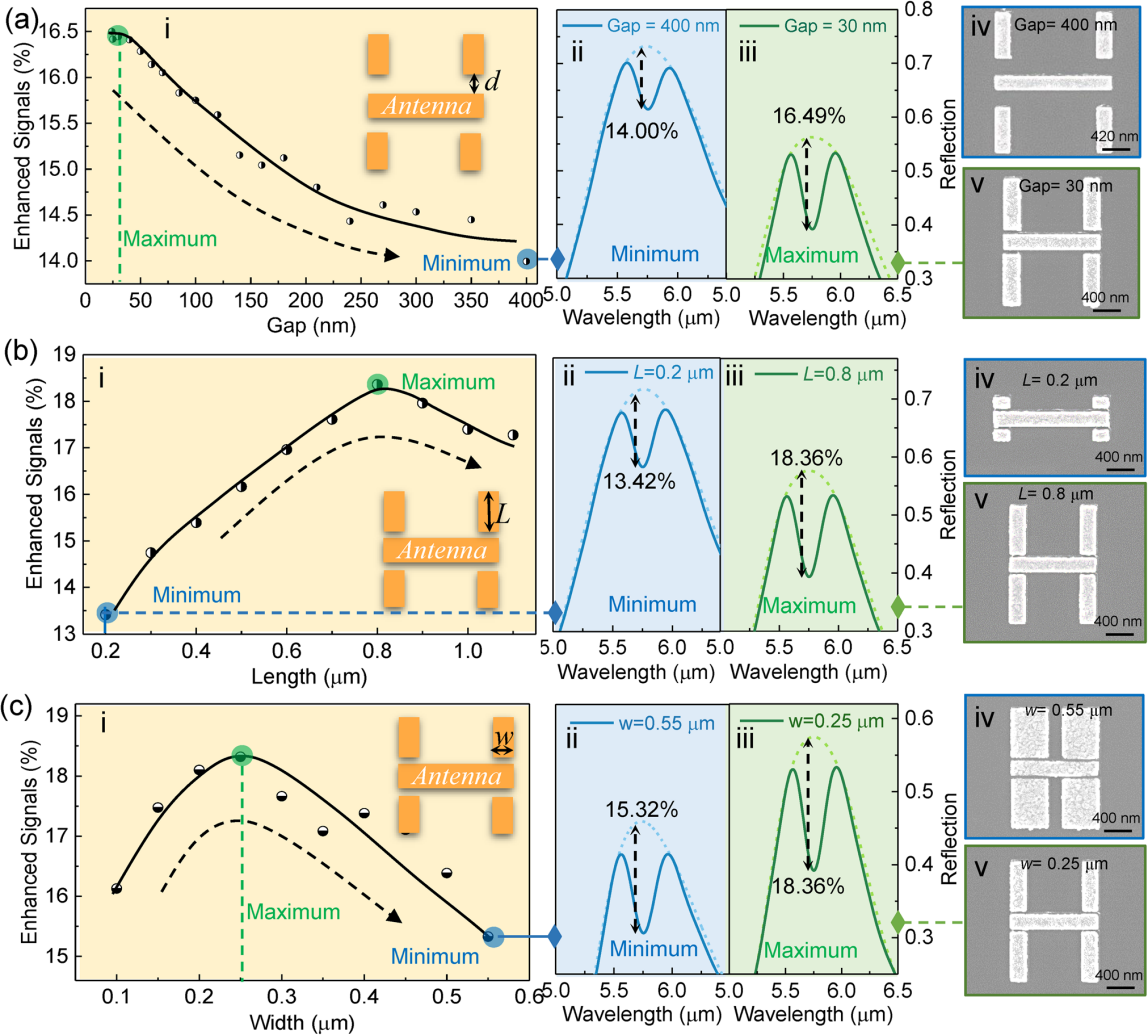
Dimension optimization of dark mode antennas in multi-hotspot strategy. **a** Influence of nanogap size on molecular signal enhancement (analyte: 40 nm thick PMMA). i: profile curve of signal enhancement versus nanogap size *d*; ii: simulated spectrum corresponding to minimum signal enhancement; iii: simulated spectrum corresponding to maximum signal enhancement; iv, v: SEM image. **b** Influence of dark mode antenna length *L* on molecular signal enhancement. **c** Influence of dark mode antenna width *w* on molecular signal enhancement. Dotted line: the baselines of spectra

The dimension optimization of the dark mode antenna is the other task for further investigation by controlling variables. It includes the optimization of nanogap size, antenna length, and width. Similar to the nanogap position, the nanogap size also affects the mode coupling efficiency and thus the molecular signal enhancement. When keeping other variables constant, the enhanced molecular signal gradually increases as the nanogap size decreases from 400 to 30 nm, as shown in Fig. 3a-i. However, the continued reduction in the nanogap saturates the growth of enhanced molecular signals. It is because the coupling efficiency achieved by 30 nm nanogap is sufficient to completely couple the dark mode antenna to the bright mode antenna. Therefore, considering the expensive nanofabrication cost of making a 10 nm nanogap, the size of the nanogap is finally set to 30 nm. Figure 3a-ii-v shows the details of spectra and SEM images corresponding to the minimum and maximum signal enhancement. The length of the bright mode antenna is fine-tuned to ensure the frequency matching between PMMA molecular vibrations and the antenna resonance. After the determination of nanogap size, we further investigate the dimension of the dark mode antenna. In general, large-size antennas have high losses. As the length *L* of dark mode antennas increases, the enhanced molecular signal undergoes an evolution that first rises and then falls (Fig. 3b-i). At the maximum signal (*L* = 8 μm), it is clear that the loss rate of the antenna system also reaches the best according to Fig. 1d, and further loss increases (via *L*) will reduce the loss rate and molecular signal. Therefore, the length *L* is finally set to 8 μm. Figure 3b-ii-v shows the details of spectra and SEM images corresponding to the minimum and maximum signal. After the determination of length, we further investigate the width *w* of the dark mode antenna. As observed in Fig. 3c, the influence of antenna width on molecular signal enhancement is similar to that of antenna length due to their similar effect on the area of the dark mode antenna. Clearly, a width of 0.25 μm achieves maximum molecular signal enhancement. When the width reaches 0.55 μm, the dark mode nanoantennas are close to each other (Fig. 3c-iv). Despite the proximity, the coupling between adjacent dark mode nanoantennas is weak. Notably, methods of controlling variables can only achieve local optimization. In our case, however, it is clear from the theoretical analysis (Fig. 1d) that the trajectory between the enhanced signal and the loss rate resembles a parabola. Therefore, the vertex of the analogous parabola that we found by continuously increasing the loss is the maximum value of the enhanced signal. Furthermore, there are multiple sets of different antenna dimensions corresponding to the maximum signal. More specifically, since the length and width of the antenna jointly determine the area of the dark mode antenna, multiple sets of antenna dimensions corresponding to the maximum signal are obtained by reducing the antenna length and increasing the antenna width simultaneously.

### Signal Enhancement Characterization of Nanoantenna-based Transduction

**Fig. 4:**
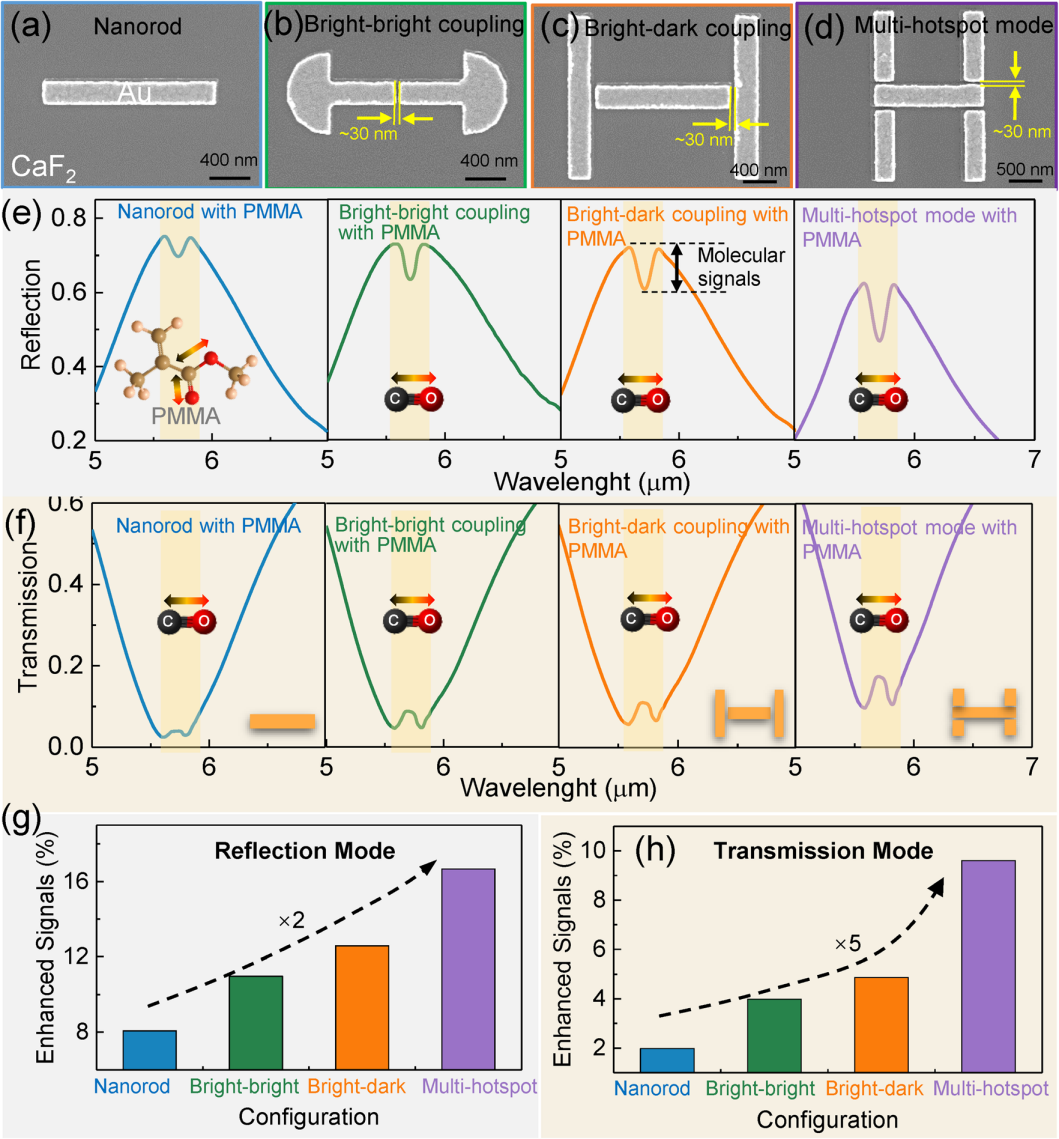
Characterization of vibrational signal enhancement. **a** SEM images showing the details of nanorods, **b** bright–bright mode coupled antennas, **c** bright–dark mode coupled antennas, and **d** multi-hotspot bright–dark mode coupled antennas. All nanogaps size is set as 30 nm. **e** Measured reflection and **f** transmission spectra of the above-mentioned nanoantennas with 40-nm-thick PMMA as analytes. **g** Corresponding enhanced molecular signal of the above-mentioned nanoantennas extracted from reflection or **h** transmission spectra

In this section, we fabricate some representative antennas with various configurations and then characterize their signal enhancement performance by spin coating a PMMA film as the analyte (see Experimental Section for details). Figure 4a-d shows the SEM images of nanorods, bright–bright mode coupled antennas, bright–dark mode coupled antennas, and multi-hotspot bright–dark mode coupled antennas. Their nanogap sizes are set to be the same, and their resonances are also all engineered to perfectly match the vibration of PMMA by fine-tuning the bright mode antenna arm length. The measured reflection and transmission spectra of these nanoantennas are shown in Fig. 4e-f. As observed, the antenna resonances are well matched to the C = O stretching vibrations of PMMA film, demonstrating the high accuracy of our fabrication process. Notably, there is a discrepancy between measured and simulated reflection spectra, which are caused by the geometrical differences between the simulated model and the fabricated antenna (Note S10). To compare the signal enhancement achieved by these antennas, the enhanced molecular signals are extracted from these spectra by using a least-squares fitting, as shown in Fig. 4g-h. As observed, multi-hotspot nanoantennas possess the highest signal enhancement in both reflection and transmission modes when compared with dual-hotspot configurations like nanorods and mode-coupled antennas. It demonstrates the effectiveness of our loss-enhanced multi-hotspot strategy. Specifically, in the reflection mode, the enhanced molecular signal of the multi-hotspot nanoantenna is 2 times higher than that of the nanorods. In transmission mode, the enhanced signal of the former is 5 times higher than that of the latter. The reason for different levels of enhancement is that the sensitivity of the antenna to the loss rate is different in reflection mode and transmission mode, as shown in Fig. 1d. In transmission mode, the intensity of enhanced molecular signals is low when the loss rate *γ*_*r*_/*γ*_*a*_ deviates from the optimal (*γ*_*r*_/*γ*_*a*_ > 2). Therefore, the improvement in the *γ*_*r*_/*γ*_*a*_ to the signal intensity is significant. However, in reflection mode, the enhanced molecular signal remains high intensity when the loss rate deviates from the optimal (*γ*_*r*_/*γ*_*a*_ > 2), so the improvement in the *γ*_*r*_/*γ*_*a*_ to the signal intensity is relatively small. The high signal intensity over a large range indicates the strong robustness of the reflection mode. Notably, when loss rate *γ*_*r*_/*γ*_*a*_ > 1, the enhancement at reflection mode is larger than that at transmission mode, and the opposite is true when *γ*_*r*_/*γ*_*a*_ < 1. For most common antennas, the *γ*_*r*_/*γ*_*a*_ is greater than 2 (*γ*_*r*_/*γ*_*a*_ of nanorod is about 10) due to the large radiation losses in comparison with absorption losses. In loss optimization to achieve the optimal *γ*_*r*_/*γ*_*a*_, the loss coupled to nanoantenna is fewer in reflection mode than in transmission mode. The less coupled to the antenna system means less attenuation of near-field intensity in reflection mode. Therefore, the reflection mode of the multi-hotspot antennas is adopted as the working mode of signal transduction of MOF hybrids for the gas detection demonstration.

### Modification and Characterization of MOFs

**Fig. 5:**
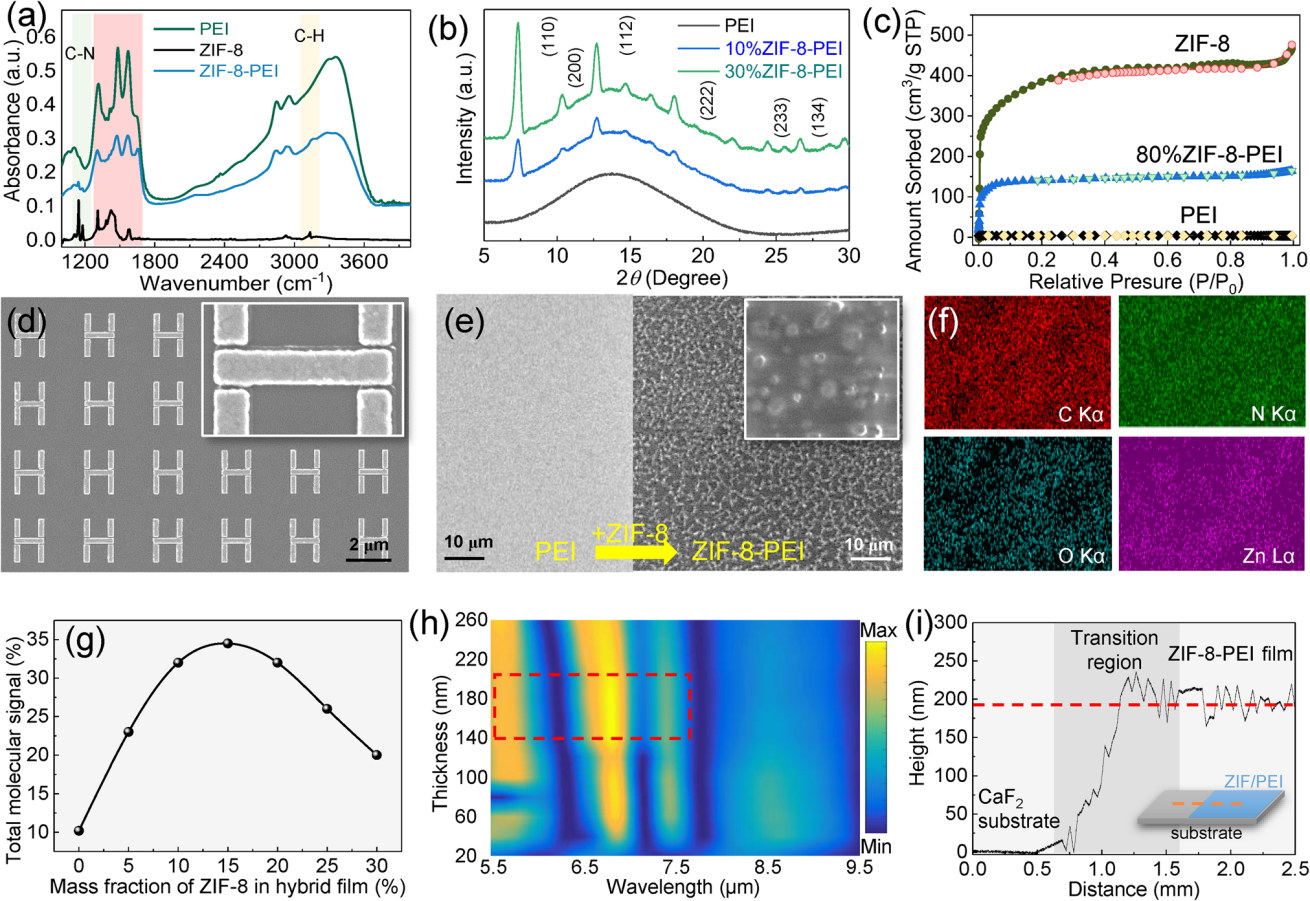
Material characterization. **a** IR absorbance spectra of PEI, ZIF-8, and ZIF-8-PEI. **b** XRD spectra of PEI, 10% ZIF-8-PEI, and 30% ZIF-8-PEI showing their phase details. **c** N_2_ adsorption–desorption isotherms for PEI, ZIF-8, and ZIF-8-PEI at 77 k showing their BET surface area. **d** SEM micrograph showing the details of our multi-hotspot nanoantennas. **e** SEM images showing the nanoantennas with PEI film (left panel) and ZIF-8-PEI hybrid film (right panel). **f** EDX mapping analysis of the ZIF-8-PEI hybrid film. **g** Profile curve of enhanced molecular signals versus ZIF-8 mass fraction showing the influence of ZIF-8 mass fraction on enhanced signals. **h** Hybrid film thickness versus spectrum wavelength map revealing the effect of film thickness on enhanced molecular signals of our multi-hotspot nanoantennas. **i** Thickness measurement of ZIF-8-PEI hybrid film using a profilometer

Enhancing the gas-induced variations in MOFs is critical for the gas detection application. In our strategy, MOFs are modified by PEI polymers to achieve the dual mechanism of physical and chemical adsorption and maximize the gas-induced changes in MOFs. Additionally, low-molecular-weight branched PEI (average M_w_ ~ 800) is chosen due to the smooth penetration of short-chain PEI into the relatively narrow pore window of MOF (ZIF-8). To investigate the composition of hybrid films and the interactions between PEI and ZIF-8, Fourier transform infrared spectroscopy (FT-IR) analysis, X-ray diffraction (XRD) test, and energy-dispersive X-ray spectroscopy (EDX) mapping, SEM, and N_2_ adsorption/desorption analysis are performed. As shown in Fig. 5a, the representative IR peaks near 1150 and 3140 cm^−1^ corresponding to *ν*(CN) and *ν*(CH) stretching vibrations of ZIF-8 are observed in the ZIF-8-PEI hybrid film, indicating the successful loading of ZIF-8 [74]. Notably, a slight blue-shift of IR peaks in amine groups between 1300 ~ 1700 cm^−1^ (red region in Fig. 5a) is observed due to the interactions from the C-N vibration of ZIF-8. It means that the hybrid film is not a simple mixture of ZIF-8 and PEI. There are some interactions of chemical bonds between them. From the perspective of Bragg diffraction angles (Fig. 5b), with the loading of ZIF-8 into PEI, sharp peaks representing the ZIF-8 crystalline structure appear in the measurement results. Moreover, the intensity of these peaks is largely increased as the ZIF-8 loading increases. It indicates that the ZIF-8 is not only successfully loaded but also preserves its crystalline structure. The N_2_ adsorption/desorption isotherms of PEI at 77 K before and after ZIF-8 loading are characterized to investigate the change in the Brunauer–Emmett–Teller (BET) surface area. As shown in Fig. 5c, the original PEI exhibits quite low N_2_ uptakes, and after the loading of ZIF-8, the N_2_ uptake, BET surface area, and pore volume increase sharply. For instance, the saturated N_2_ uptake of 80% ZIF-8-PEI hybrid film reaches 419 cm^3^ g^−1^, and the BET surface area is calculated to be 406 m^2^ g^−1^ by applying the Brunauer–Emmett–Teller equation. The high surface area of the hybrid film ensures the contact area between gases and films, thereby optimizing the spatial overlapping of molecules to the near field.

In addition to the compositional changes, the effect of MOF hybrids on the morphology of the device is also huge. For nanoantennas fabricated by electron beam lithography (EBL) and evaporation (see Experimental Section), the morphology and outline are clear and well defined (Fig. 5d). After the loading of gas enrichment, the nanoantenna pattern is no longer observed in the SEM image (Fig. 5e). When the gas enrichment material is pure PEI film, the surface morphology of the device is smooth and dense (left panel in Fig. 5e), which is not friendly to the adsorption of gas molecules. When the gas enrichment material is the PEI-modified ZIF-8 film, rough surfaces with ZIF-8 particles inside are observed (right panel in Fig. 5e), which is conducive to the diffusion of gas into the film. Notably, the crystal size of the synthesized ZIF-8 is large (over 200 nm), and the penetration depth of the near field of the antenna is generally less than 200 nm (Note S11). Therefore, to address their size mismatch, the ZIF-8 crystals are sonicated to make them small and uniform (Note S12). The EDS results in Fig. 5f demonstrate the uniform distribution of ZIF in PEI, where the purple-labeled Zn element is what distinguishes ZIF-8 from PEI. The content of each element of ZIF-8-PEI hybrid films is shown in Note S13. In addition to the morphology distribution of ZIF-8, its mass fraction also has a critical effect on the enhanced molecular signal achieved by the antenna system. As observed in Fig. 5g, the content of 15% ZIF-8 in the hybrid film is suitable for the nanoantenna. Either high or low content will reduce the detection performance of the nanoantenna. It is because the hybrid film with low ZIF-8 content is unfavorable for gas diffusion, while the hybrid film with high ZIF content leads to low gas adsorption due to the lack of reactive adsorption sites for gas in PEI. After the determination of the MOF mass fraction, we investigate the influence of film thickness on the enhanced molecular signal. Considering that the resonance of the nanoantenna will be red-shifted thanks to the increase in film thickness, while the IR vibration position of the molecule is fixed, the length of the bright mode antenna is finely tuned to avoid detuning. Figure 5h shows the map of enhanced molecular signal when film thickness and spectrum wavelength change. As observed, the IR peaks in ZIF-8-PEI are distinct in the map, and with increasing film thickness, the enhanced molecular signal between 5.5 and 7.5 μm first increases and then decreases slightly. Gas enrichment materials with an unsuitable thickness are not beneficial for gas adsorption. According to the signal enhancement profile, the sensor with 180 nm thick enrichment exhibits the highest performance. Therefore, 180 nm is selected as the optimal value of the film thickness. By adjusting the rotation speed and time during the spin coating process, the film thickness can be controlled. Based on the measurement results in Fig. 5i, the final thickness of the hybrid film is about 185 nm.

### Demonstration of Ultrasensitive Gas Detection

**Fig. 6:**
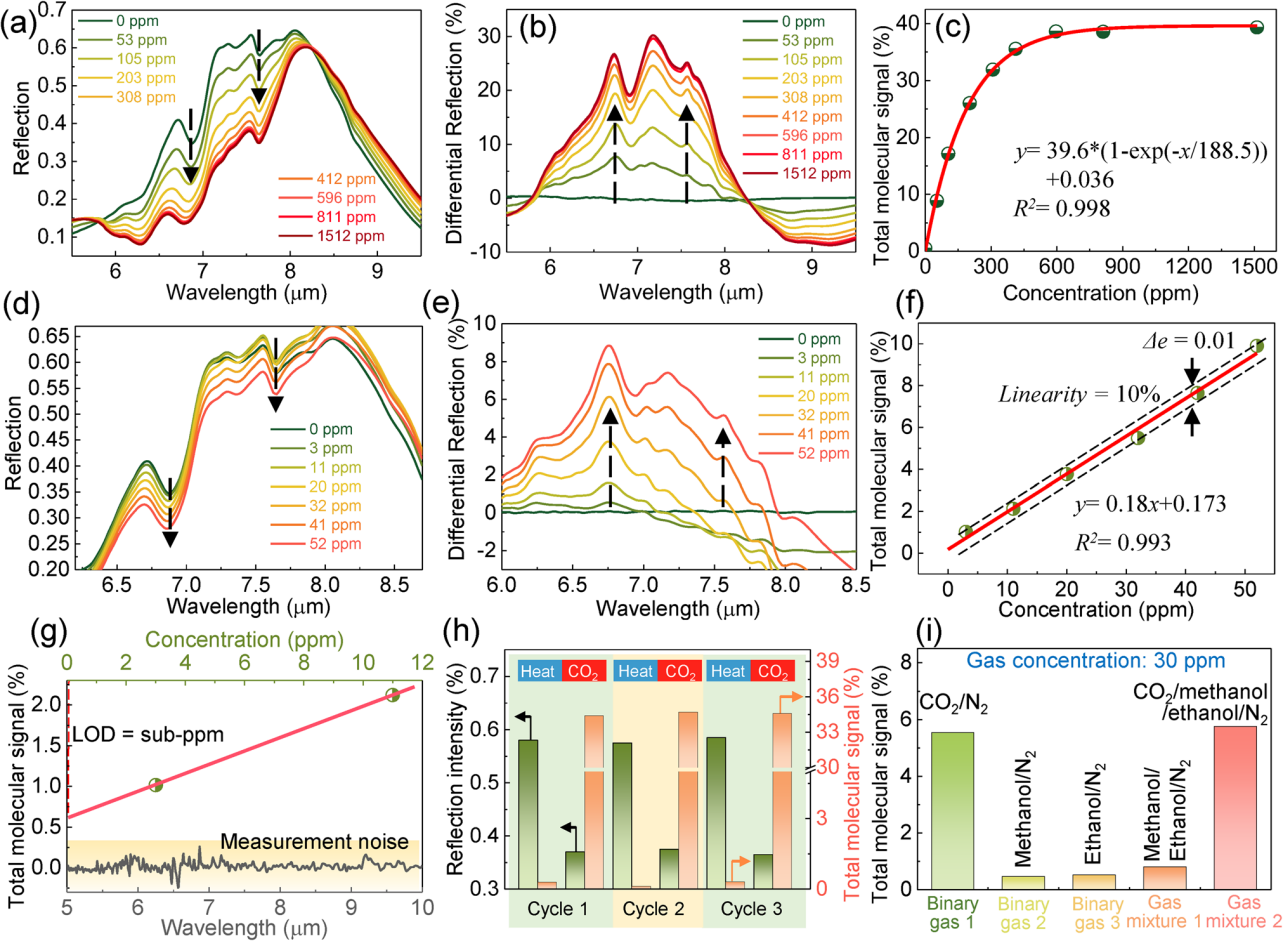
Demonstration of ultrasensitive CO_2_ gas detection using the MOF-functionalized multi-hotspot platform. **a** Measured spectral response of the hybrid platform when CO_2_ concentrations vary over a wide range (0–1512 ppm). **b** Corresponding differential signal with the measured spectrum of 0 ppm as reference. **c** Total molecular signal versus CO_2_ concentration profile showing the sensing behavior of the platform over a wide concentration range. **d** Measured spectral response of the platform in a small CO_2_ concentration range (0–52 ppm). **e** Corresponding differential signal. **f** Total molecular signal versus CO_2_ concentration profile in a small CO_2_ concentration range. **g** Limit of detection (LOD) of the hybrid platform for CO_2_ gas detection. **h** Thermal recovery and repeatability of the platform. The reflection intensity and total molecular signal change repeatedly when the platform undergoes consecutive cycling between thermal desorption and CO_2_ loading. **i** Selectivity of the platform for CO_2_ gas detection. When measuring different gas mixtures (N_2_ as carrier gas), the signal response of the platform is significant in the presence of CO_2_ gas

Steady and dynamic responses are critical metrics for gas sensors [75–77]. Figure 6a shows the measured spectral response of the hybrid platform after reaching steady state at various concentrations. As observed, significant changes occur in the 6–8 μm band of the IR spectrum, which is due to the group changes in the MOF-PEI hybrids caused by the adsorption of CO_2_. They correspond to N–H deformation in NH_3_ ^+^ , C = O stretching, NCOO skeletal vibration, and the conformational change in HCO_3_^-^ (Note S3). To observe the changes clearly, the differential reflection spectra are extracted by taking the spectrum without CO_2_ as a reference (Fig. 6b). Apparently, the intensity of differential reflection rises with the increase in CO_2_ concentration. To consider all gas-induced spectral changes, the differential reflection spectra in the 6–8 μm band are calculated by integration as the total molecular signal (see Experimental Section). Figure 6c depicts the total molecular signal versus CO_2_ concentration profile over a wide concentration range (0–1512 ppm). As observed, with increasing CO_2_ gas concentration, the total molecular signal undergoes an evolution that first sharply increases and then gradually reaches saturation. Their nonlinear relationship is similar to the Langmuir isotherm model for gas adsorption, reflecting the critical role of the MOF-PEI hybrid in sensing behavior. When CO_2_ varies within a small range of ultra-low concentrations (0–52 ppm), the spectral response of the platform is similar (Fig. 6d-e). However, the relationship between the total molecular signal and CO_2_ gas concentration becomes linear (linearity: 10%), as shown in Fig. 6f. The reason is that the reactive adsorption sites for gas molecules in the MOF hybrids are completely sufficient at low concentrations. When the slope of the fitting curve is defined as the sensor sensitivity, it reaches 0.18%/ppm, which is about 45 times higher than that using pure MOFs (Note S14).

The limit of detection (LOD) is a crucial figure of merit for evaluating the sensing behavior of the platform and is closely related to noise. Total noise is obtained by acquiring the spectra of the platform at pure nitrogen (N_2_) gas 20 times and then extracting and averaging the signal fluctuations of these spectra. By plotting the total noise and the output signal of the platform at low concentrations together, we can analyze the LOD of the platform (Fig. 6g). As observed, the signal strength of our platform at a CO_2_ gas concentration of 0 ppm is still higher than the total noise of the measurement system. The margin of the signal above the noise at 0 ppm indicates that the LOD of our platform could reach ppb levels. However, owing to the limitations of the experimental setup (difficult to find commercial ppb-level CO_2_ sensors), the detailed performance of our platform in the ppb concentration range has not been investigated. To the best of our knowledge, 1 ppm LOD is the highest among infrared CO_2_ sensors (Table S2). The low detection limit enables some new applications in industry, such as CO_2_ monitoring in hydrogen production. Apart from LOD, the reversibility of the platform is also investigated. When the platform undergoes consecutive cycling between CO_2_ inflow and thermal desorption (Fig. 6h), the measured spectral peak (pine green) and total molecular signal (orange) of the platform change accordingly. The difference between all adsorption and desorption cycles is within 2%. The difference is due to the noise from the spectral collection of nanoantennas. The noise includes white noise and other hybrid noise. The white noise is a discrete random noise caused by the measurement system and device. In addition to white noise, there are other hybrid noises in the measurement such as the spectral shift caused by the change in environmental air composition and the thermal noise of the device. The noise could be reduced by background calibration or combining machine learning algorithms. In addition to the reversibility, we also investigate the selectivity of the platform (Fig. 6i). Apparently, the signal response of the platform is significant in the presence of CO_2_ gas, and in the absence of CO_2_ gas, the signal response is weak. The excellent selectivity is attributed to the strong interaction of CO_2_ with amines and the molecular size confinement of MOFs.

**Fig. 7:**
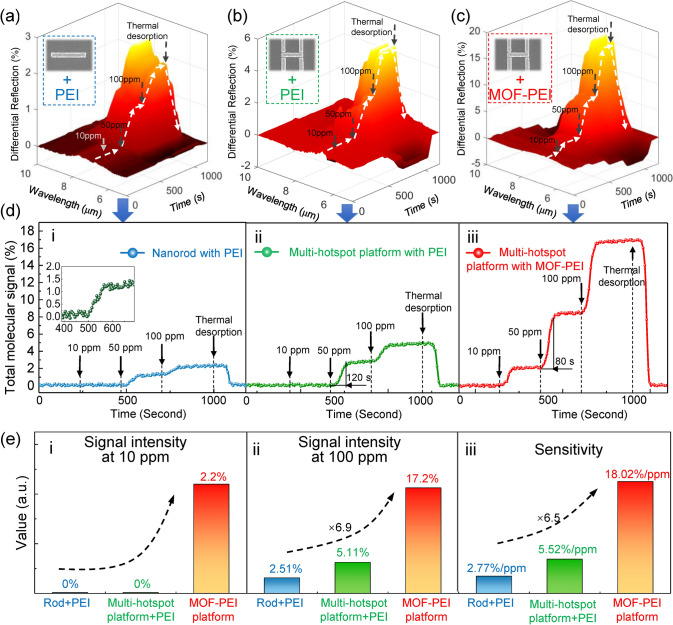
Dynamic behavior of sensing platforms. **a** Real-time 3D plots of the differential spectra of platforms using PEI-coated nanorods, **b** PEI-coated multi-hotspot antennas, and **c** MOF-PEI-functionalized multi-hotspot antennas, when CO_2_ gases with various concentrations are loaded and thermally desorbed. **d** Temporal variation in the total molecular signal corresponding to (**a-c**), revealing the dynamic behavior of various platforms. i: PEI-coated nanorod platform; ii: PEI-coated multi-hotspot platform; iii: MOF-PEI-functionalized multi-hotspot platform. **e** Performance comparison corresponding to (**d**). i: spectral signal intensity of platforms after loading 10 ppm CO_2_ gas; ii: spectral signal intensity after loading 100 ppm CO_2_ gas; iii: sensitivity comparison

The dynamic behavior of the platform is investigated by placing it in a gas cell with a heating stage inside, and the gas concentration of the cell is controlled in real time by mass flow controllers (see Experimental Section). The spectral response of the platform is recorded every 5 s. To investigate the advantages of MOF and multi-hotspot design in gas detection, platforms using PEI-covered nanorods and PEI-covered multi-hotspot antennas are set as the control group, and the platform using MOF-PEI-functionalized multi-hotspot antennas is the experimental group (Fig. 7a-c). When the gas concentration in the gas cell is sequentially subjected to 0 ppm, 10 ppm, 100 ppm, and thermal desorption, significant changes in the differential spectra of platforms are observed. By calculating the differential spectra as total molecular signals, it is clear that the response of different platforms to concentration changes is distinct (Fig. 7d). When the CO_2_ gas concentration is 10 ppm, no response is observed in the PEI-functionalized platform, while the response of the MOF-PEI-functionalized platform is significant. It indicates that the integration of physisorption and chemisorption greatly improves the detection limit (Fig. 7e-i). When the CO_2_ gas concentration was increased to 100 ppm, all the platforms show a clear response (Fig. 7e-ii). The signal intensity of the platform using multi-hotspot antennas is more than 2 times higher than that using common nanorods, which is attributed to the coordination of contradictions by the multi-hotspot strategy. In terms of sensitivity, the MOF-PEI platform is 6.5 times higher than the nanorod platform (Fig. 7e-iii). When the response time is defined as the signal intensity reaching 95% of the stable value, the response time of the MOF-PEI platform is also 33% faster than that of the PEI-functionalized platforms (Fig. 7d). Notably, there could be a slight loss of PEI after multiple thermal cycles for a long time, which could be due to the formation of urea (amidation) between CO_2_ and amine of PEI in the gas adsorption process [78]. The loss can be reduced by adding a crosslinker to the PEI film [79]. Additionally, the long optical interaction length is a major factor hindering the miniaturization of conventional infrared gas sensors. For instance, to ensure sufficient light-matter interactions for IR detectors to recognize gas-induced changes in IR intensity, non-dispersive infrared (NDIR) sensors require an optical interaction length of at least centimeters to reach a ppm detection level limit, which makes the sensor bulky [80]. Our platform is miniaturized with an optical interaction length of only about 200 nm, which is about 37,000 times shorter than that of miniaturized NDIR sensors (7.5 mm) [81] and even shorter than commercial NDIR sensors (at least centimeters) [82]. Notably, nanoantenna-based sensing schemes do not conflict with NDIR sensing technology. There are works reporting that nanoantennas can improve the sensitivity and detection performance of NDIR [83]. Furthermore, we use a conventional infrared spectrometer to provide the light source and detector for our nanoantenna-based gas sensor demonstration, which makes the entire setup bulky. The detection system can be miniaturized in three ways, including the integration of miniaturized spectrometers [84, 85], the use of imaging-based sensor arrays [86], and detecting gases at specific wavelengths (Note S15). The comparative analysis of the performance between our platform and the existing IR CO_2_ gas sensors is listed in Table S2. Our platform has strong competitiveness among them. Compared with other kinds of CO_2_ gas sensors, our platform shows an advantage in detection limit, sensitivity, and light–matter interaction length (Note S17).

## Conclusion

In summary, we have demonstrated the extension of MOFs for ultrasensitive CO_2_ gas sensing by using nanoantennas as signal transduction. To maximally boost the sensing performance, MOFs are modified by adding amino groups to possess both physical and chemical adsorption functions. Besides, a multi-hotspot nanoantenna strategy is proposed to reconcile the optimization contradiction by utilizing the increase in hotspot number to compensate for the near-field intensity weakening, achieving near-field optimization and loss optimization simultaneously. As a result, the modified MOF-polymer hybrids capture CO_2_ gas more easily than pure MOFs, and its sensitivity as the gas-selective-trapping materials of the platform reaches 0.18%/ppm, which is 45 times higher than that of pure MOFs. Furthermore, by co-designing and optimizing the position of the nanogap and the dimension of the dark mode antenna, our multi-hotspot nanoantennas have a five-fold higher molecular signal enhancement than the common nanorod antennas. Benefiting from pioneering efforts in MOFs modification and nanoantenna optimization, we demonstrate the competitive advantages of our strategy in state-of-the-art CO_2_ gas sensors, including low LOD, high sensitivity (0.18%/ppm), excellent reversibility (variation within 2%), high selectivity, and nm-level optical interaction length for miniaturization. This work gains new insights into the integration of advanced porous materials and nanophotonic devices, as well as opens the door to some harsh industrial applications such as ultra-low concentration CO_2_ monitoring in hydrogen production.

## Supplementary Information

Supplementary Notes S1−S17, including overview of optimization methods; gas detection setup; spectral change of MOF PEI hybrids before or after CO_2_ gas absorption; general temporal coupled mode theory analysis; electric field distribution and intensity during position optimization processes; IR spectrum of PMMA; number of hotspots for different antenna configurations; SEM images and measured spectra of antennas during position optimization; loss extraction using TCMT; comparison between simulation and measurement; analysis of near field penetration depth of the platform; SEM analysis of MOF; EDX analysis of MOF; sensitivity of platform with various configurations; platform miniaturization; performance comparison; comparison with electrochemical and photoacoustic CO_2_ sensors.

## Supplementary Information

Below is the link to the electronic supplementary material.

Supplementary file1 (PDF 1621 KB)
