# Supplementary material for: MOF/Polymer-Integrated Multi-Hotspot Mid-Infrared Nanoantennas for Sensitive Detection of CO2 Gas
Source: Nanomicro Lett. 2022 Oct 22;14:207. doi: 10.1007/s40820-022-00950-1 (PMC9588146; doi:10.1007/s40820-022-00950-1)
Supplement: Supplementary file 1 — Supplementary file1 (PDF 1621 KB) [file 40820_2022_950_MOESM1_ESM.pdf]

Supporting Information for

MOF/Polymer-Integrated Multi-Hotspot Mid-Infrared Nanoantennas for Sensitive Detection of CO_2 Gas

Hong Zhou[1, 2, #], Zhihao Ren[1, 2, #], Cheng Xu[1, 2], Liangge Xu[1, 2], Chengkuo Lee[1, 2, 3, *]

[1] Department of Electrical and Computer Engineering, National University of Singapore, 117583, Singapore

[2] Center for Intelligent Sensors and MEMS (CISM), National University of Singapore, 117608, Singapore

[3] NUS Suzhou Research Institute (NUSRI), Suzhou 215123, P. R. China

[#] Hong Zhou and Zhihao Ren contributed equally to this work.

* Corresponding author. E-mail: elelc@nus.edu.sg (Chengkuo Lee)

S1 Overview of Enhancement Optimization Methods of Nanoantennas

Infrared (IR) fingerprint vibrations are directly related to the molecular components, chemical bonds, and their conformations. The detection of fingerprint vibrations in infrared spectroscopy provides great opportunities for the non-invasive and non-destructive identification of various substances in nature. However, it has encountered a technical bottleneck of low sensitivity due to the weak light-molecule interaction in the mid-IR (~10^{-20} cm^2 per molecule), which limits its application in the detection of trace molecules, such as trace gas sensing and monolayer molecular monitoring. Resonant nanoantenna is an effective solution to enhance such light-molecule interaction by exciting collective oscillations of electrons at the surfaces. Although the concept of light-molecule interaction enhanced by nanoantennas is well-established, the optimization of nanoantennas to maximally strengthen the interaction is critical and still presents a challenge. Currently, there are three methods of enhancing the plasmon-molecule interactions in the nanoantenna sensing technology. These methods include i) increasing the spatial overlapping of molecules to the near-field, ii) enhancing near-field intensity, and iii) loss optimization, as shown in Fig. S1 and Table S1.

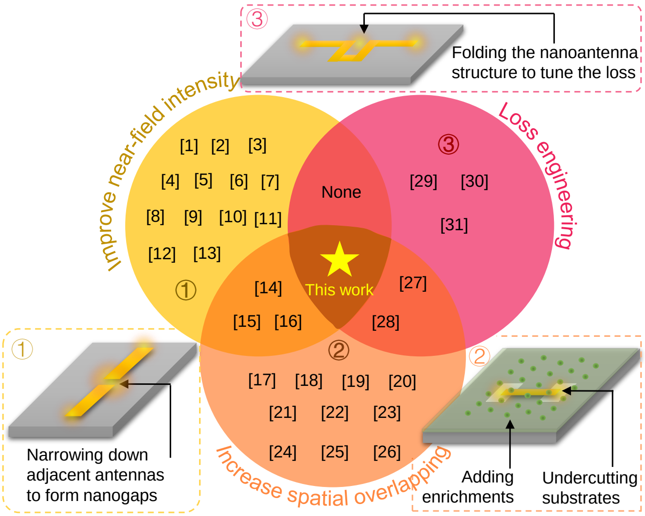

Fig. S1 Paper review showing the overview of optimization methods of enhancing the plasmon-molecule interactions using infrared nanoantennas. Improving near-field intensity only [S1-S13], Improving both loss engineering and spatial overlapping [S14-S16], increasing spatial overlapping only [S17-S26], both overlapping improvement and loss engineering [S27-S28], and loss engineering only [S29-S31].

S1.1 Increasing the Spatial Overlapping of Molecules to the Near-field

Better spatial overlap means more analytes are located in the enhanced near-field, resulting in higher plasmon-molecule interactions. There are mainly two schemes that have been proven effective. Approaches from the antenna design side are to free up effective sensing space for molecules by undercutting the antenna structure or integrating microfluidic channels with the dielectric substrate. For instance, the enhanced electromagnetic field of the metal-insulator-metal (MIM) nanoantenna scheme is mainly concentrated in the dielectric spacer. Therefore, in our previous work, we integrate the microfluidic channel with the dielectric spacer in a metal-insulator-metal nanoantenna scheme, where the channel functions as both the microfluidic channel for sample delivery and the spacer for isolating antennas and reflection metal layer [S27]. In this configuration, the analytes in the microfluidic channel had an excellent spatial overlap with the enhanced electromagnetic field. Additionally, the near-field of the antenna is exposed as much as possible in free space by undercutting the antenna to facilitate molecular coupling. In addition, it is also effective to undercut the antenna so that the near field of the antenna is exposed in free space as much as possible [S22]. Then, more molecules can enter the exposed near field to increase the spatial overlapping. The method from the molecule side is the introduction of enrichment materials in the near-field to concentrate molecules [S26]. Enrichment materials are critical for molecule detection at low concentrations, especially gas molecules that are distributed loosely and flow freely in free space.

S1.2 Enhancing Near-field Intensity of Nanoantennas

The widely used approach to increase the near-field intensity is the proximity of adjacent nanoantennas to form nanogaps, where strong near-field interaction occurs between the adjacent nanoantennas due to their capacitive coupling [S3]. Additionally, the addition of an expansion structure at the end of the antenna (opposite to the gap) to increase the charge storage capacity can further improve the localized near-field intensity [S7]. For instance, a broadband log-periodic trapezoidal nanoantenna with a minimum gap of 20 nm between units was fabricated by using the EBL method [S1]. It was demonstrated that the enhancement factor of this design reached 0.9×10^5, and the detection of alkanethiol molecules with attomolar concentrations ($<1.2\times10^6$ molecules) was achieved. When the gap was narrowed down to 3 nm, the enhancement factor was increased to 10^7 and 600 molecules of 4-NTP were successfully detected. That is, the enhancement factor is enhanced by two orders of magnitude when the minimum gap is reduced from 20 to 3 nm. Although the method is effective, continually narrowing down the nanogap for pursuing higher enhancement optimization is costly due to the requirement of high-precision fabrication.

S1.3 Loss Engineering/Optimization

The plasmon-molecule interaction in the antenna is realized by the coupling of antenna resonance and molecular vibration. The total loss in a plasmon-molecule coupled system can be calculated as $\gamma_{total} = \gamma_r + \gamma_a$. The loss rate γ_r/γ_a will change when external radiation γ_r or intrinsic material absorption γ_a are tuned. Therefore, there are two kinds of methods to tune the loss rate. From the perspective of γ_r, it represents the loss of energy during the coupling of the plasmonic mode to the propagating light. One effective way to tune γ_r is folding the structure of nanoantennas while keeping the absorptive loss almost constant by not changing the total area of the nanoantenna. For instance, in our previous work, we demonstrated that the bending degree of the antenna is inversely related to γ_r via developing a crooked nanoantenna [S28]. It was demonstrated that Molecule signals were increased by 25 times without sub-100 nm structures. In terms of γ_a, it describes the intrinsic absorption of materials, that is, Ohmic loss of metal nanoantennas. An effective way to tune γ_a is adding a metal reflection layer close to

nanoantennas, thereby forming a well-known perfect absorber. Our previous work demonstrated that γ_r and γ_a were changed by tuning the distance between the reflection layer and the nanoantenna [S27].

S1.4 Integration of Multiple Optimization Methods

To maximally strengthen the plasmon-molecule interactions, it is of great significance to integrate multiple optimization methods. As shown in Fig. S1 and Table S1, the combination of near-field improvement and overlapping optimization has been reported many times, as has the integration of loss engineering and overlapping optimization. However, the integration of near-field optimization and loss engineering has not been experimentally exploited in vibrational spectroscopy. As stated in the main text, it is due to the contradiction between nearfield optimization and loss engineering. That is, the improvement achieved by near-field optimization could be attenuated by loss optimization due to the loss increment. This contradiction has been demonstrated in Fig. 1g of the main text. As observed, the average near-field intensity of antennas using the loss engineering method is lower than that of antennas using nearfield optimization. Our multi-hotspot strategy can complement the weakened near-field and achieve the highest near-field intensity. More specifically, the nanogap is designed beside the hotspot of bright mode antennas to excite new high-intensity hotspots and compensate for the near-field intensity weakening caused by loss optimization, thereby achieving near-field optimization and loss optimization simultaneously. Together with the optimization of the spatial overlapping achieved by MOF enrichments, this work enables the integration of all major optimization methods used in antenna-based plasmon-molecule coupling systems.

Table S1 Optimization methods of enhancing the plasmon-molecule interaction using infrared nanoantennas

Methods	Refs.	Detailed optimization methods	Wavelength region
Near-field intensity improvement	Aouani et al. [S1]	Using multi-frequency gold optical antennas with narrow nanogap for ultrasensitive broadband probing of molecular vibrational modes	2.5-10 μm
	Yoo et al. [S2]	Developing coaxial zero-mode resonators with sub-10 nm gaps for protein detection	4-10 μm
	Ji et al. [S3]	Fabricating a metamaterial absorber with sub-5 nm gaps for mid-infrared absorption spectroscopy	2-5 μm
	Dong et al. [S4]	Using a bowtie-shaped Au structure with a sub-3 nm gap for monolayers of molecule detection	5.5-8.3 μm
	Brown et al. [S5]	Developing four rods oriented at right angles with a small gap at the central junction	2.5-5 μm
	Metzger et al. [S6]	Positioning ITO nanocrystals into the hot-spot of plasmonic gap-antennas to enhance performance	7.1-14.2 μm
	Brown et al. [S7]	Utilizing fan-shaped gold nanoantennas above reflective substrates for sensitive vibration detection	2.1-6.6 μm
	Yue et al. [S8]	Using multiple-resonant pad-rod nanoantennas to improve the near-field intensity	3-7 μm
	Mackin et al. [S9]	Utilizing the arms of trimeric antennas to form nanogap for near-field intensity enhancement	4.5-10 μm

Methods	Refs.	Detailed optimization methods	Wavelength region
	Semenyshyn et al. [S10]	Using the resonant plasmonic nanoslits formed by nanorod antennas for sensitive molecular detection	5-7.1 μm
	Chen et al. [S11]	Using waveguide-integrated antennas with a 30 nm gap for mid-infrared spectroscopic chemical sensing	2.7-4 μm
	John-Herpin et al. [S12]	Using grating order-coupled nanogap antennas for sensitive biomolecule detection	6.02-6.17 μm
	Chen et al. [S13]	Employing a modified "Sketch and Peel" lithography fabricate narrow nanogaps for intensity enhancement	5.8-9 μm
Both near filed and overlapping improvement	Hwang et al. [S14]	Fabricating a vertical 10 nm nanogap between antennas and reflection, which enhances both near-field intensity and overlapping	2.5-5 μm
	Hwang et al. [S15]	Using bottom-up techniques to fabricate a vertical nanogap to enhance near-field and overlapping	2.5-3.6 μm
	Jung et al. [S16]	Developing a vertical nanogap for both near-field intensity and overlapping enhancement	1.8-2 μm
Overlapping improvement	Su et al. [S17]	Developing antennas with the vertically oriented structure to improve spatial overlapping	3-4 μm
	Zhou et al. [S18]	Integrating porous metal-organic framework with antenna for gas sensing	5.5-12.5 μm
	Le et al. [S19]	Integrating a nanofluidic channel with the dielectric substrate to improve spatial overlapping	2.9-4.2 μm
	Zhou et al. [S20]	Integrating porous metal-organic framework with dual-resonant antennas for gas detection	3-9 μm
	Zhou et al. [S21]	Using polyvinylamine as enrichment to concentrate gas molecules and improve overlapping	4-8 μm
	Cetin et al. [S22]	Undercutting nanoantennas to improve spatial overlapping and sensitive biosensing	1.4-2 μm
	Le et al. [S23]	Integrating a nanofluidic channel with the dielectric substrate to improve spatial overlapping	2-5 μm
	Chang et al. [S24]	Developing an all-dielectric surface-enhanced infrared absorption-based gas sensor with enrichment	5.5-7 μm
	Chong et al. [S25]	Integrating porous metal-organic framework with plasmonic antennas for gas detection	2.6-2.8 μm
	Bareza et al. [S26]	Developing boron nitride nanoantennas with polyethylenimine enrichment for gas detection	6.6-7 μm
Overlapping improvement and loss optimization	Xu et al. [S27]	Integrating channels with the dielectric substrate and adjusting the gap distance for loss optimization	2-4 μm
	Wei et al. [S28]	Integrating polyethylenimine enrichment and tuning loss by crooking nanoantennas at the same time	5-7 μm
Loss optimization	Ren et al. [S29]	Hooking nanoantennas to tune the loss for sensitive detection and identification of alcoholic molecules	5-9 μm
	Adato et al. [S30]	Investigating loss mechanisms in coupled molecular and plasmonic resonator systems	5-7 μm

Methods	Refs.	Detailed optimization methods	Wavelength region
	Wei et al. [S31]	Crooking nanoantennas to tune the loss for sensitive sensing applications	3-8 μm
All integrated	This work	A multi-hotspot nanoantenna strategy to reconcile the contradiction of methods to achieve near-field and loss optimization simultaneously. At the same time, using physi-chemisorption of MOFs for overlapping improvement.	5-8 μm

S2 Gas detection Setup

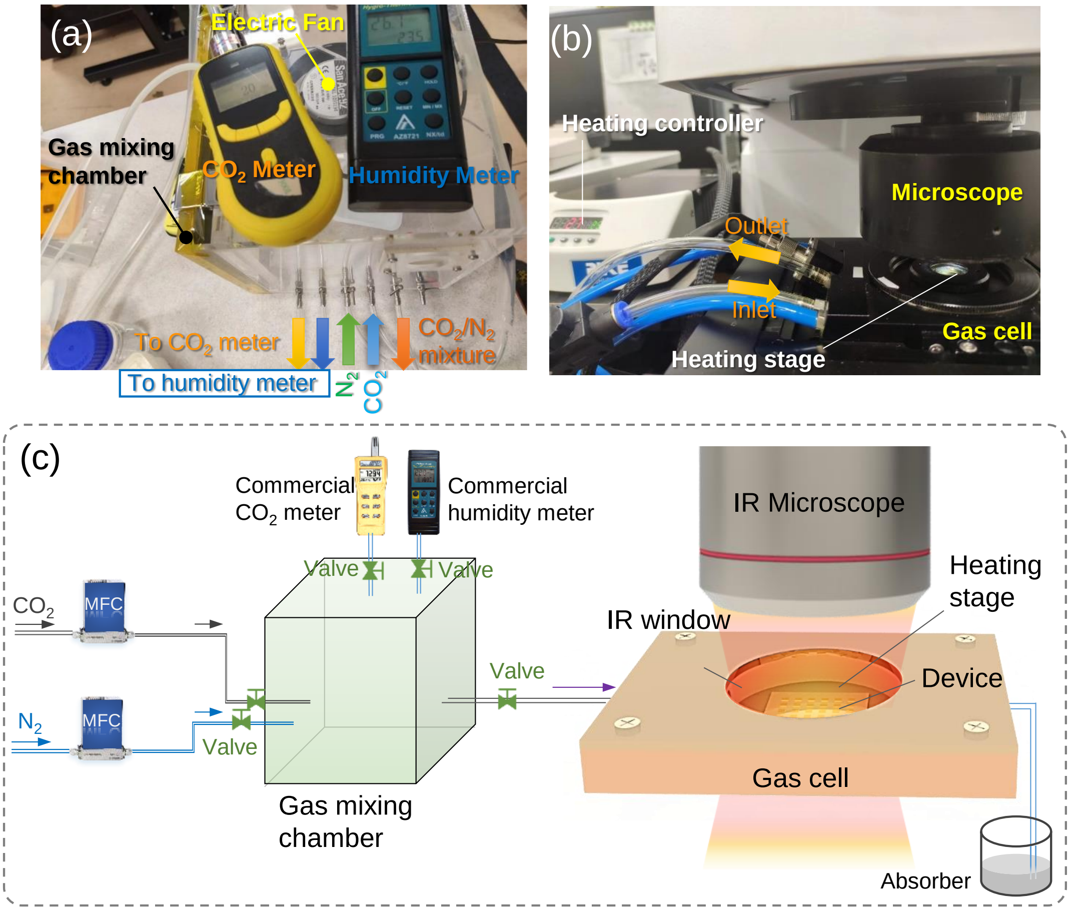

Fig. S2 Gas detection setup. **a** The photo of gas mixing system. **b** The photo of gas cell and IR microscope. **c** Schematic diagram of the gas detection setup. First, the target CO_2 gas or N_2 gas enters the mixing chamber through gas mass flow controllers (MFC), which were adjusted in real time to obtain the desired gas flow rates. Then, the gas flowing out of the MFC is quickly mixed uniformly by a fan in the mixing chamber. After mixing, the gas flowed into the gas cell where our platform was located. The gas cell consists of upper and lower IR barium fluoride windows, a sample holder, and a heating stage, where the sample holder and heating stage are integrated to efficiently heat our sensing platform

S3 Spectral Change of MOF-PEI Hybrids Before or After CO_2 Gas Absorption

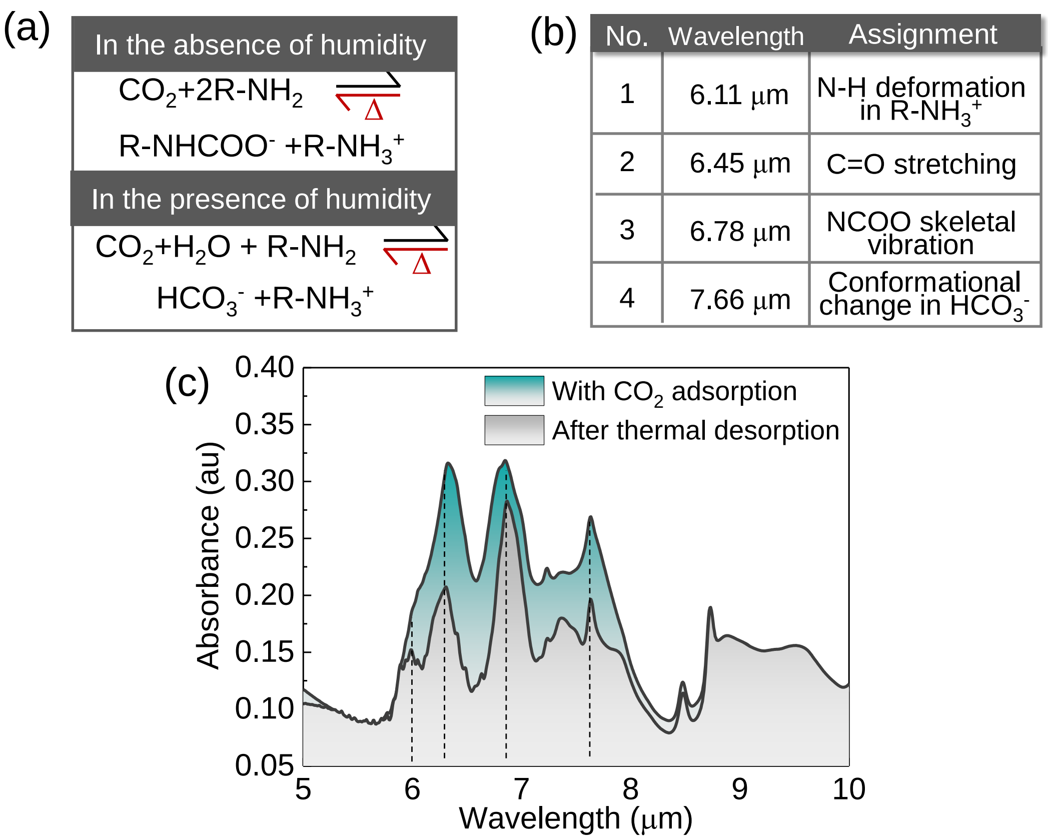

Fig. S3 Spectral change of MOF-PEI hybrids before or after CO_2 gas absorption. **a** Chemical reactions related to the adsorption and desorption of CO_2 in the MOF-PEI hybrids. **b** The associated wavelength assignments for the primary by-products of the reaction path. **c** Spectral change of MOF-PEI hybrids before or after CO_2 gas absorption

S4 General Temporal Coupled-mode Theory Analysis

The loss optimization of nanoantennas is based on general temporal coupled-mode theory (TCMT). As shown in Fig. S4, the TCMT describes a single cavity representing the bright mode (A) of the nanoantennas coupled (κ) with two ports including incident light (S_{1+}), reflected light (S_{1-}), and transmitted light (S_{2-}). It experiences damping at a rate γ_r and γ_a, and the molecular damping γ_m is coupled to the nanoantennas via a coupling strength μ. The TCMT analysis consists of three processes, including modeling the bare nanoantennas, modeling the nanoantennas coupled with molecules, and modeling the enhanced gas sensing signal of our platform.

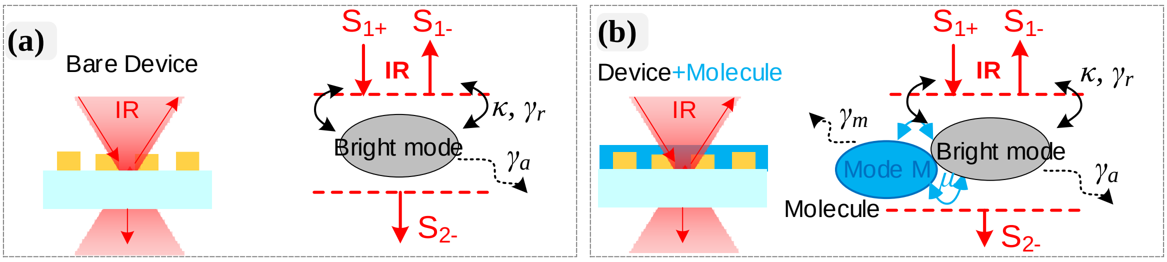

Fig. S4 Schematic of TCMT. **a** Bare nanoantennas. **b** Nanoantennas coupled with molecules

S4.1 Modeling the Bare Nanoantennas

According to TCMT, a series of equations describing the interaction between antenna and IR can be obtained as

$$\frac{dA}{dt} = j\omega_0 A - (\gamma_\mathrm{r} + \gamma_\mathrm{a}) A + \kappa S_{1+} \tag{S1}$$

$$S_{2-} = S_{1+} - \kappa A \tag{S2}$$

$$S_{1-} = \kappa A \tag{S3}$$

$$R = \left| \frac{S_{1-}}{S_{1+}} \right|^2 \tag{S4}$$

$$T = \left| \frac{S_{2-}}{S_{1+}} \right|^2 \tag{S5}$$

Since the nanoantenna is a dual-port resonance system, we can obtain $\kappa = \sqrt{\gamma_r}$. Then, Equation S1 can be simplified as

$$A = \frac{\sqrt{\gamma_r} S_{1+}}{j(\omega - \omega_0) + \gamma_a + \gamma_r} \tag{S6}$$

By substituting Equation S6 to Equation S2-S5, the reflection and transmission of bare nanoantennas can be written as

$$T_{bare} = \frac{(\omega - \omega_0)^2 + \gamma_a^2}{(\omega - \omega_0)^2 + (\gamma_a + \gamma_r)^2} \tag{S7}$$

$$R_{bare} = \frac{\gamma_r^2}{(\omega - \omega_0)^2 + (\gamma_a + \gamma_r)^2} \tag{S8}$$

As observed, the reflection and transmission at resonance (ω=ω_0) are determined by the loss of the coupling system. When defining $f = \gamma_r/\gamma_a$, the reflection and transmission of bare nanoantennas can be expressed as

$$T_{bare} = \frac{f^2}{(1+f)^2} \tag{S9}$$

$$R_{bare} = \frac{1}{(1+f)^2} \tag{S10}$$

Equations S9 and S10 are used to analyze the spectral performance of bare nanoantennas, and the plotted results are shown in Fig. 1d of the main text.

S4.2 Modeling the Nanoantennas Coupled with Molecules

When molecules are loaded to the nanoantennas, the lossy molecular vibration (denoted as M) is coupled (coefficient μ) to the bright-mode nanoantenna as a purely dissipative mode. Then, the coupled mode equations in this configuration can be written as

$$\frac{dA}{dt} = j\omega_0 A - (\gamma_\mathrm{r} + \gamma_\mathrm{a}) A + j\mu M + \kappa S_{1+} \tag{S11}$$

$$\frac{dM}{dt} = j\omega_\mathrm{m} M - \gamma_\mathrm{m} M + j\mu A \tag{S12}$$

Equations S11 and S12 can be simplified as

$$\left[j\left(\omega-\omega_0\right)+\left(\gamma_\mathrm{r}+\gamma_\mathrm{a}\right)\right]A=j\mu M+\kappa S_{1+} \tag{S13}$$

$$\left[j\left(\omega-\omega_\mathrm{m}\right)+\gamma_\mathrm{m}\right]M=j\mu A \tag{S14}$$

By substituting Equations S14 to S13, we can eliminate M and obtain

$$\left[j\left(\omega-\omega_0\right)+\left(\gamma_\mathrm{r}+\gamma_\mathrm{a}\right)+\frac{\mu^2}{j\left(\omega-\omega_m\right)+\gamma\mathrm{m}}\right]A=\kappa S_{1+} \tag{S15}$$

By substituting Equation S15 to Equations S2-S5, the reflection and transmission of nanoantennas coupled with molecules can be written as

$$T(\omega)=\left|\frac{j(\omega-\omega_0)+\gamma_a+\dfrac{\mu^2}{j(\omega-\omega_m)+\gamma_m}}{j(\omega-\omega_0)+(\gamma_a+\gamma_r)+\dfrac{\mu^2}{j(\omega-\omega_m)+\gamma_m}}\right|^2 \tag{S16}$$

$$R(\omega)=\left|\frac{\gamma_r}{j(\omega-\omega_0)+(\gamma_a+\gamma_r)+\dfrac{\mu^2}{j(\omega-\omega_m)+\gamma_m}}\right|^2 \tag{S17}$$

Equations S16 and S17 is used to extract absorptive and radiative loss of nanoantennas coupled with molecules by fitting the calculated spectrum to the resonance spectrum. In addition, when μ=0, that is, the bare nanoantenna case without coupled molecular, we can find that $T(\omega)$ and $R(\omega)$ in Equation S16 and S17 are the same with T_{bare} and R_{bare} in Equation S7 and S8, which proves the consistency of our derivation.

S4.3 Modeling the Enhanced Gas Sensing Signal

The enhanced molecular signal of our platform in sensing application is analyzed by calculating the differential spectra of the nanoantennas with/without coupled molecules. Notably, several assumptions are required in the following derivation to acquire relatively simple analytical results. First, the sensing system is in a non-detuned state. That is, the bright-mode antenna is fine-tuned to ensure the frequency matching between molecular vibrations and the antenna resonance, which is in line with our actual sensing demonstration. Second, μ is set as a much smaller parameter compared with γ_m, γ_r, and γ_a. Therefore, the enhanced signal from reflection spectrum caused by the adsorption of CO_2 can be calculated by

$$\Delta R\left(\omega=\omega_0\right)=R_{\omega=\omega_0=\omega_m}-R_{bare}=\left|\frac{\gamma_r}{(\gamma_a+\gamma_r)+\dfrac{\mu^2}{+\gamma_m}}\right|^2-\frac{\gamma_r^2}{\left(\gamma_a+\gamma_r\right)^2}$$

$$=\frac{-\gamma_r^2\,\mu^2/\gamma_m}{(\gamma_a+\gamma_r+\mu^2/\gamma_m)\left(\gamma_a+\gamma_r\right)}\square\frac{2\gamma_r^2\left(\gamma_a+\gamma_r\right)+\gamma_r^2\,\mu^2/\gamma_m}{\left(\gamma_a+\gamma_r+\mu^2/\gamma_m\right)\left(\gamma_a+\gamma_r\right)}$$

$$=-\frac{2\mu^2}{\gamma_m}\square\frac{\gamma_r^2}{\left(\gamma_a+\gamma_r+\dfrac{\mu^2}{\gamma_m}\right)\left(\gamma_a+\gamma_r\right)}\,\underline{\underline{\mu\square\gamma_m}}\,-\frac{2\mu^2}{\gamma_m}\square\frac{\gamma_r^2}{\left(\gamma_a+\gamma_r\right)^3}$$

$$\underline{\underline{f=\gamma_r/\gamma_a}}-\frac{2\mu^2}{\gamma_a\gamma_m}\frac{f^2}{(1+f)^3} \quad \text{(S18)}$$

Similarly, we can obtain the enhanced signal from the transmission spectrum as

$$\Delta T(\omega=\omega_0)=T_{\omega=\omega_0=\omega_m}-T_{bare}=\frac{2\mu^2}{\gamma_a\gamma_m}\frac{f}{(1+f)^3} \quad \text{(S19)}$$

The negative sign in Equation S18 indicates that the changes in the reflection spectrum are the opposite of the changes in the transmission spectrum. Equations S18 and S19 are used to analyze the enhanced molecular signal of our sensing platform, and the plotted results are shown in Fig. 1d of the main text. For comparison, the reflection and transmission changes are absolute values.

S5 Electric Field Distribution and Intensity During Position Optimization Processes

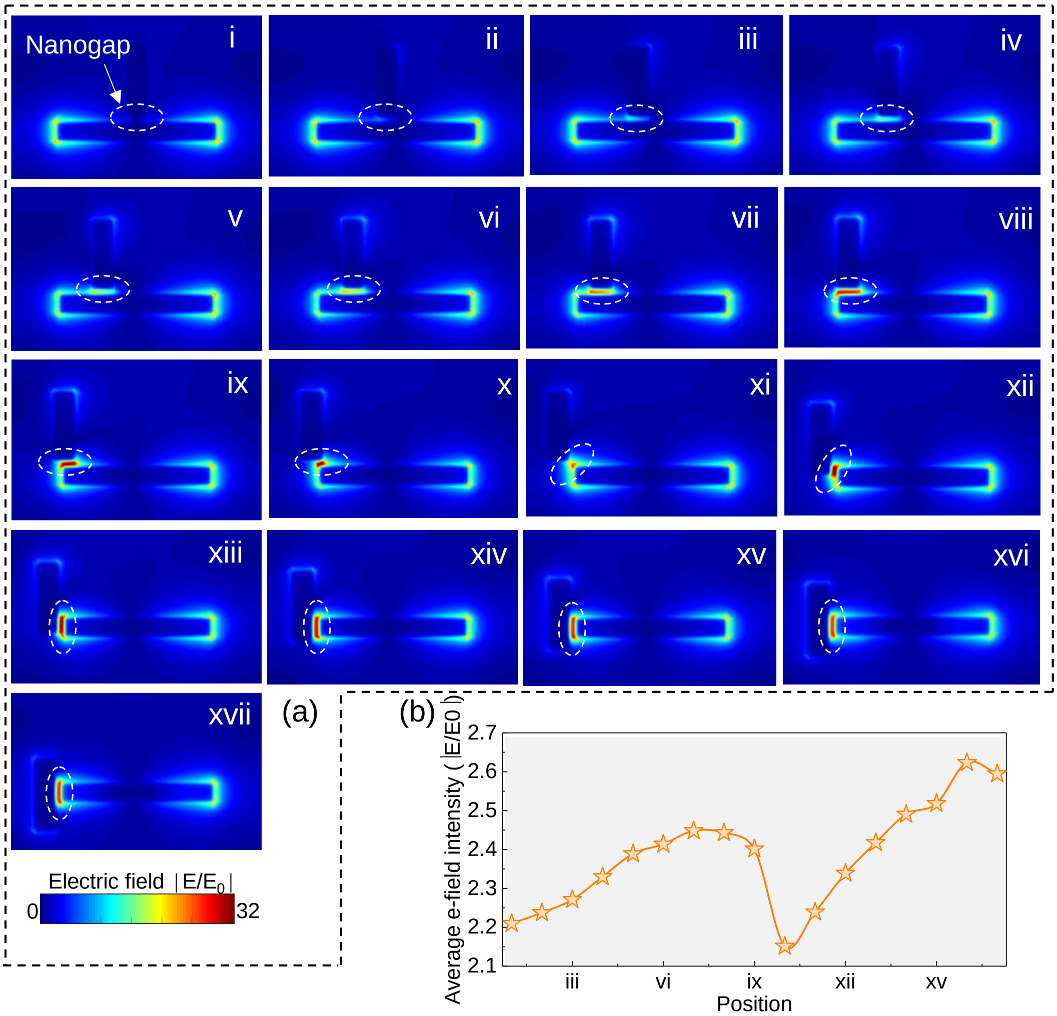

Fig. S5 Electric field distribution and intensity during position optimization processes. **a** Electric field distribution of nanoantennas when the nanogap shifts from the horizontal center to the vertical center. **b** Corresponding electric field intensity. As observed, when the nanogap shifts horizontally from the horizontal center to the end of the bright-mode antenna, the electric field intensity first increases and then decreases. When the nanogap moves vertically from the end to the vertical center, the electric field intensity undergoes a similar evolution

S6 IR Spectrum of PMMA

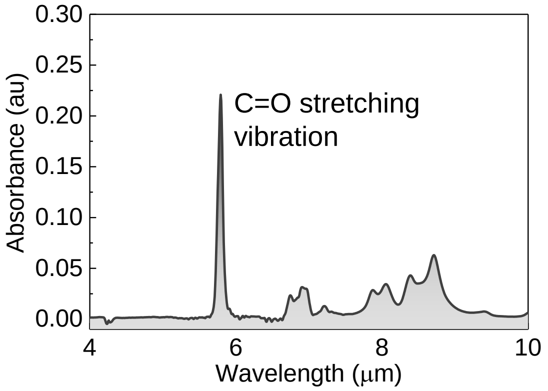

Fig. S6 IR spectrum of PMMA. As observed, there is a significant C=O stretching vibration near 5.8 μm, which is selected as the target IR vibration to investigate the performance of platform

S7 Number of Hotspots for Different Antenna Configurations

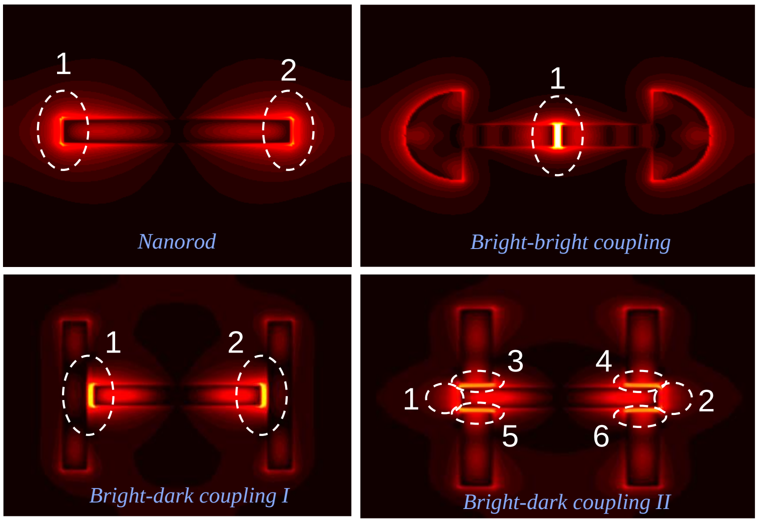

No.	Configuration	Nanogap Position	Hotspot number
1	Nanogap	NA	2
2	Bright-bright coupling	Horizontal center	1
3	Bright-dark coupling I	Vertical center	2
4	Bright-dark coupling II	End-aligned position of antennas	6

Fig. S7 Number of hotspots for different antenna configurations. Our multi-hotspot strategy can establish four new hotspots beside the two hotspots at the ends of the nanoantenna when compared with other designs. The newly established hotspots can compensate for the reduced average near-field strength in the antenna system due to increased losses of loss optimization, thereby reconciling the contradiction between near-field enhancement and loss optimization. Finally, our multi-hotspot strategy can achieve near-field optimization and loss optimization simultaneously

S8 SEM Images and Measured Spectra of Antennas During Position Optimization Processes

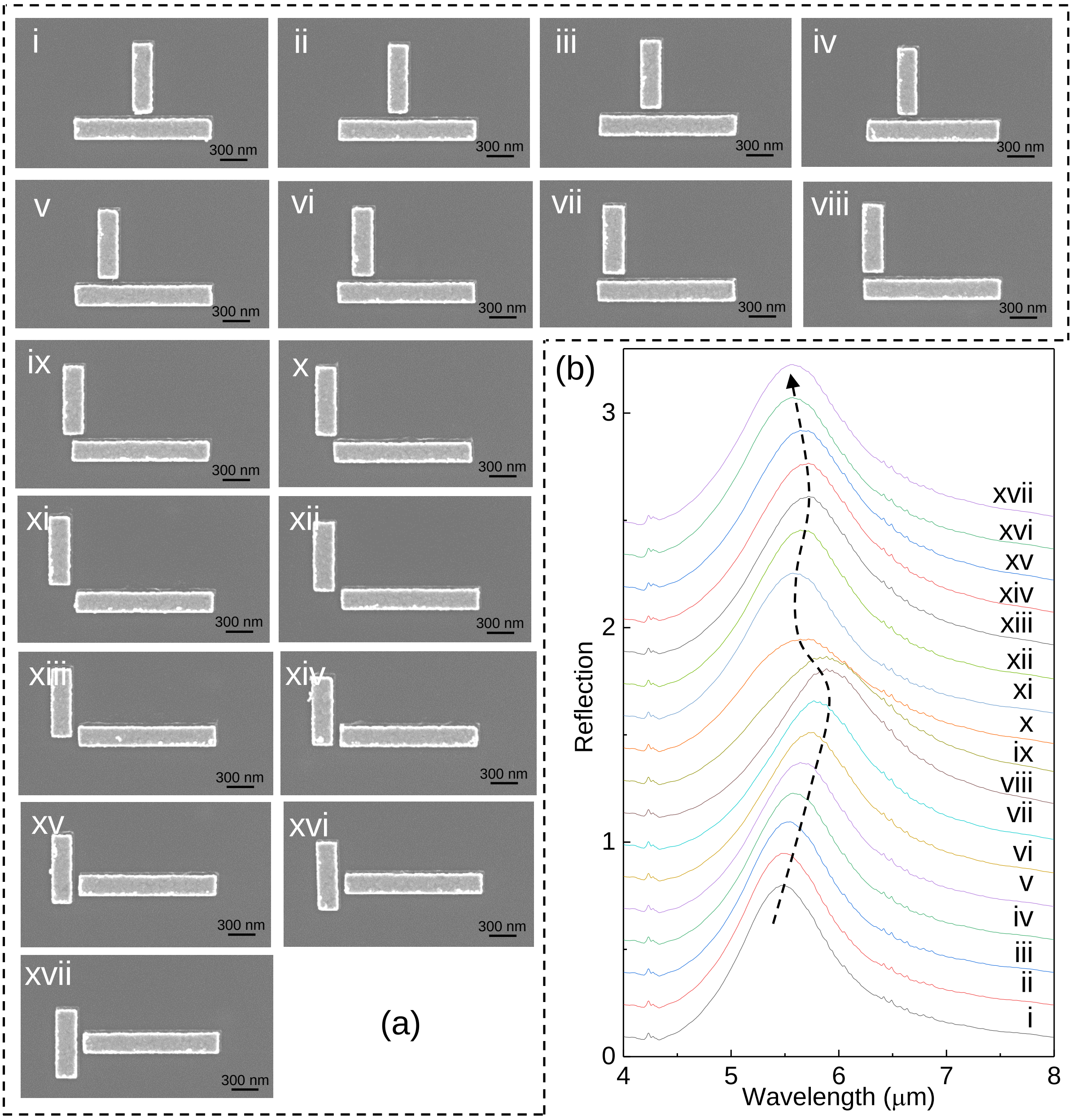

Fig. S8 SEM images and measured spectra of antennas during position optimization processes. **a** SEM images of nanoantennas when the nanogap shifts from the horizontal center to the vertical center. **b** Corresponding measured reflection spectra. As observed, the resonant wavelength of nanoantennas shifts due to the introduction of different losses. Its changing trend is the same as that of the reflection peak, and the maximum change is at position viii

S9 Loss Extraction using TCMT

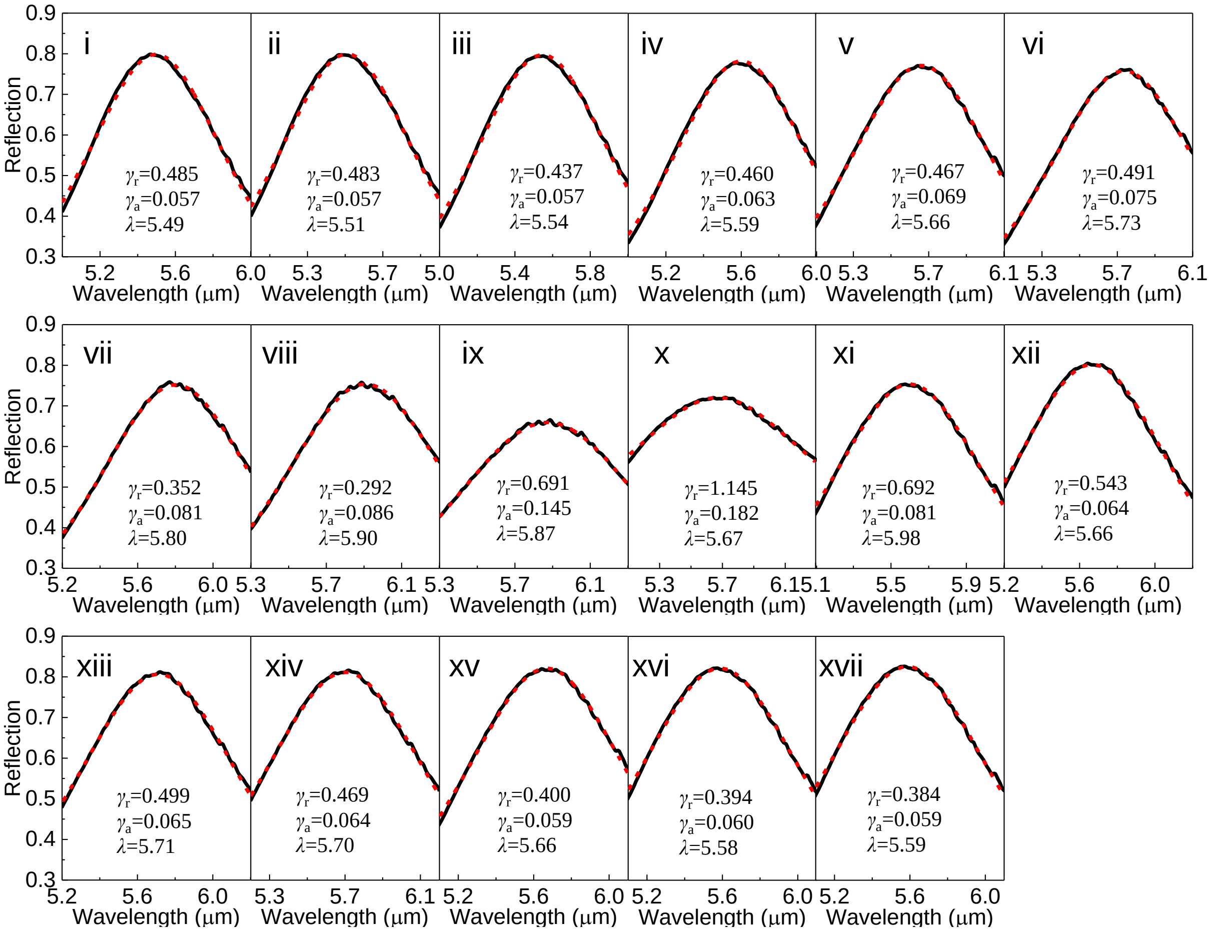

Fig. S9 Theoretical fitting of reflection spectrum of nanoantennas using TCMT. The measured spectra are from Fig. S8. Apparently, the fitting results match well with the spectrum of the platform

S10 Comparison between Simulation and Measurement

As shown in Fig. S10, there is indeed a noticeable discrepancy between the measured and simulated reflection spectra. The discrepancy is mainly concentrated in the spectral amplitude, and the resonant wavelengths are well matched. The only difference came from the geometry of the nanoantenna. The geometrical boundary of the antenna in the simulation is perfect and smooth, while the SEM in Fig. 4 of main text shows that the geometrical boundary of the fabricated antenna is not smooth and exhibits irregular undulation morphology. The irregular undulations in the antenna could affect the coupling strength of the dark mode antenna to the bright mode antenna. Due to the irregularities, it is difficult to fully replicate the shape of the fabricated nanoantenna in the simulation. Since the difference in shape mainly affects the absolute value of the spectrum, it does not affect the underlying physical mechanism. Furthermore, due to the uniformity of fabrication, its effect on all nanoantennas is consistent. The experimental results in Fig. 4 have demonstrated the effectiveness of our method. Therefore, we believe that this discrepancy does not affect the conclusion and it can be reduced by more precise fabrication methods.

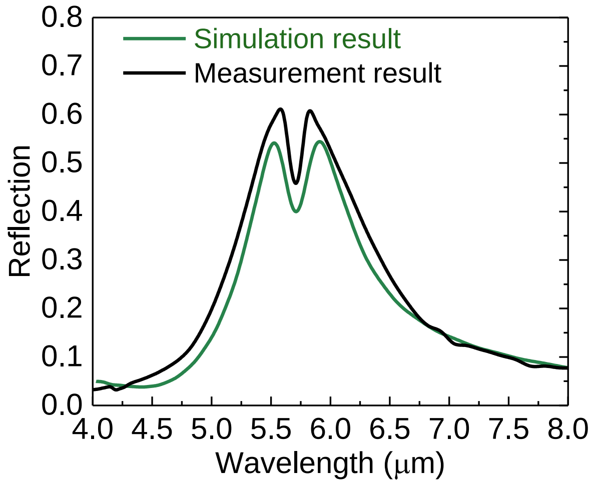

Fig. S10 Comparison between the measured and simulated reflection spectra of nanoantennas with 40 nm PMMA. The simulated result is copied from *Figure 3c-iii* of the main text. The measured result is copied from *Figure 4e* of the main text

S11 Analysis of Near-field Penetration Depth of the Platform

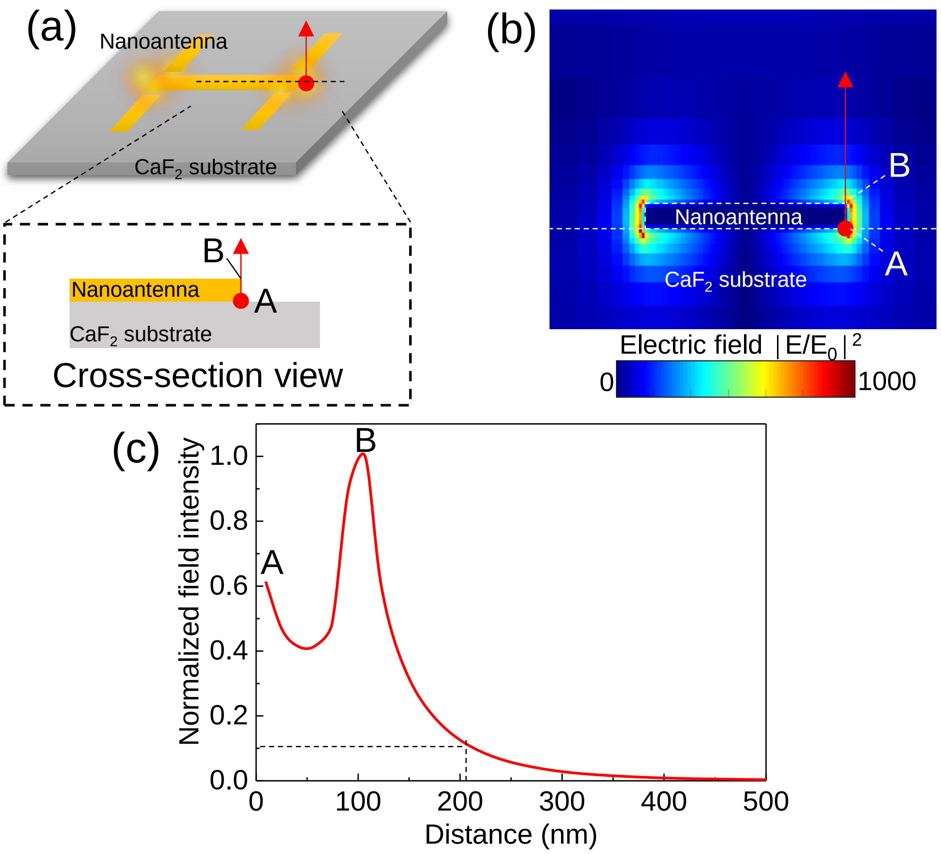

Fig. S11 Analysis of near-field penetration depth of the platform. **a** Schematic of the platform. The near-field of the cross-section at the location indicated by the dashed line is analyzed. **b** Corresponding near-field distribution of the cross-section of the platform. **c** Normalized field intensity of the cross-section of the platform as a function of the distance to the surface of the substrate (point A). As observed, when the field intensity corresponds to 10% of the maximum field strength is defined as the penetration depth, the penetration depth is about 210 nm. Variations in MOFs beyond the near-field penetration depth are difficult to detect.

S12 SEM Analysis of MOF

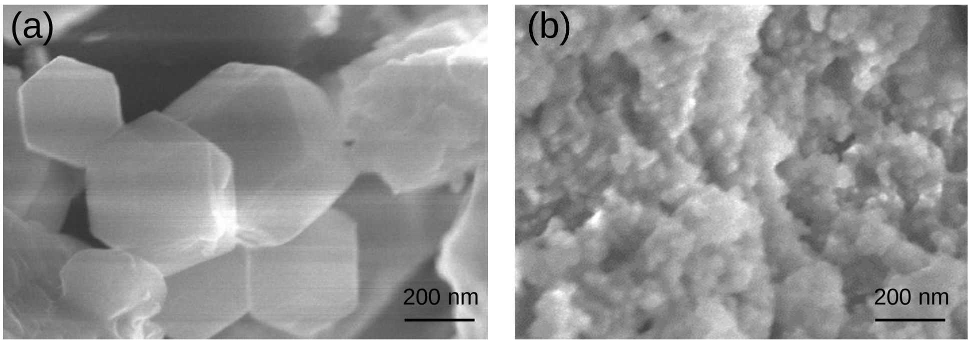

Fig. S12 SEM analysis of MOF. **a** Original crystal morphology. **b** Crystal morphology after ultrasound

S13 EDX Analysis of MOF

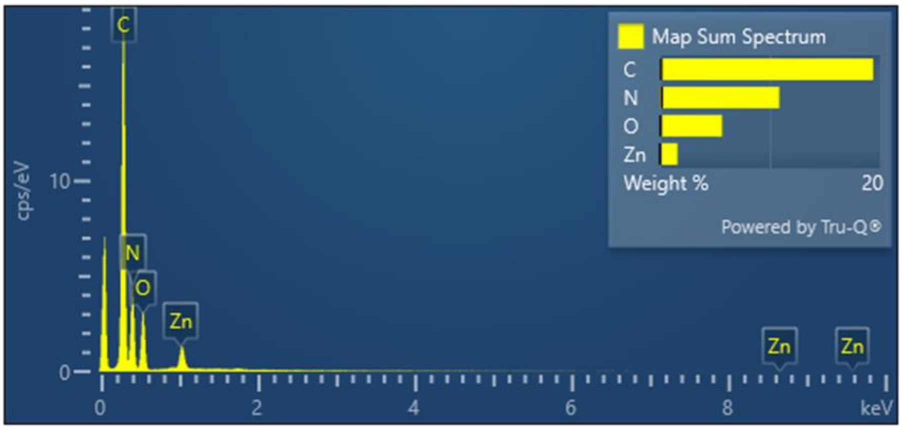

Fig. S13 EDX analysis of MOF showing the elemental composition of the MOF and its proportions.

S14 Sensitivity of Platform with Various Configuration

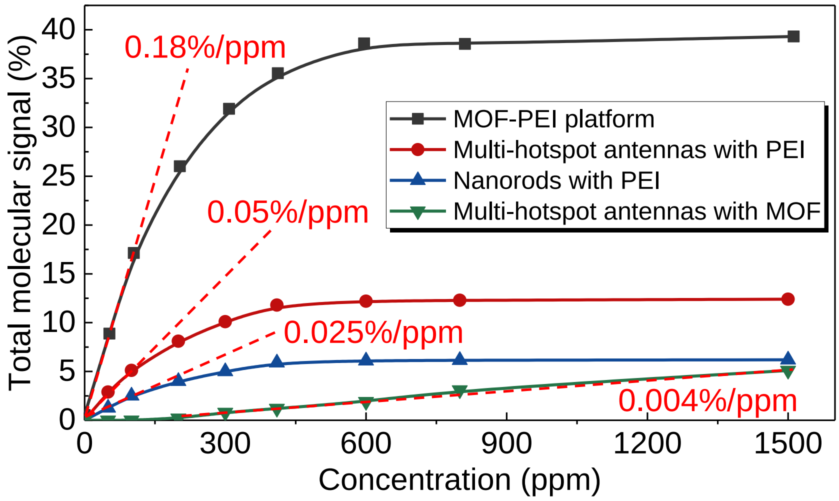

Fig. S14 Sensitivity of platform with various configurations

S15 Platform Miniaturization

There are three ways to miniaturize the system. One approach is to replace traditional bulky spectrometers with miniaturized spectrometers. The miniaturized spectrometers could be a black phosphorus spectrometer or a graphene metasurface modulator [S32, S33]. The technical challenge of this approach is the integration of two different chips. Another approach is to design nanoantennas as arrays with resonant wavelengths covering the infrared range, which can enable spectrometer-less detection via imaging [S34]. Since this method requires an external light source and detector array, its miniaturization is challenging. Besides, the output signal of the chip is multiplexed, which is also a challenge to the subsequent signal processing. Finally, we can determine the concentration of CO_2 gases by collecting infrared absorption at specific wavelengths rather than the entire infrared spectrum. Fig. S14a shows the measurement results of our sensing platform at various CO_2 concentrations, which corresponds to Fig. 5a of the main text. As observed, the relationship between the reflection intensity at 7.66 μm and gas concentration can be described by an exponential function (Fig. S14b). It means that in order to achieve miniaturization, we can integrate a conventional IR source, a detector and a 7.66 μm filter with our chips, as shown in Fig. S14c. Because our chip utilizes plasmon-molecular interactions, the optical path length required for our platform to achieve sensitive detection is small, which is an advantage for miniaturization when compared with the most widely used NDIR technology.

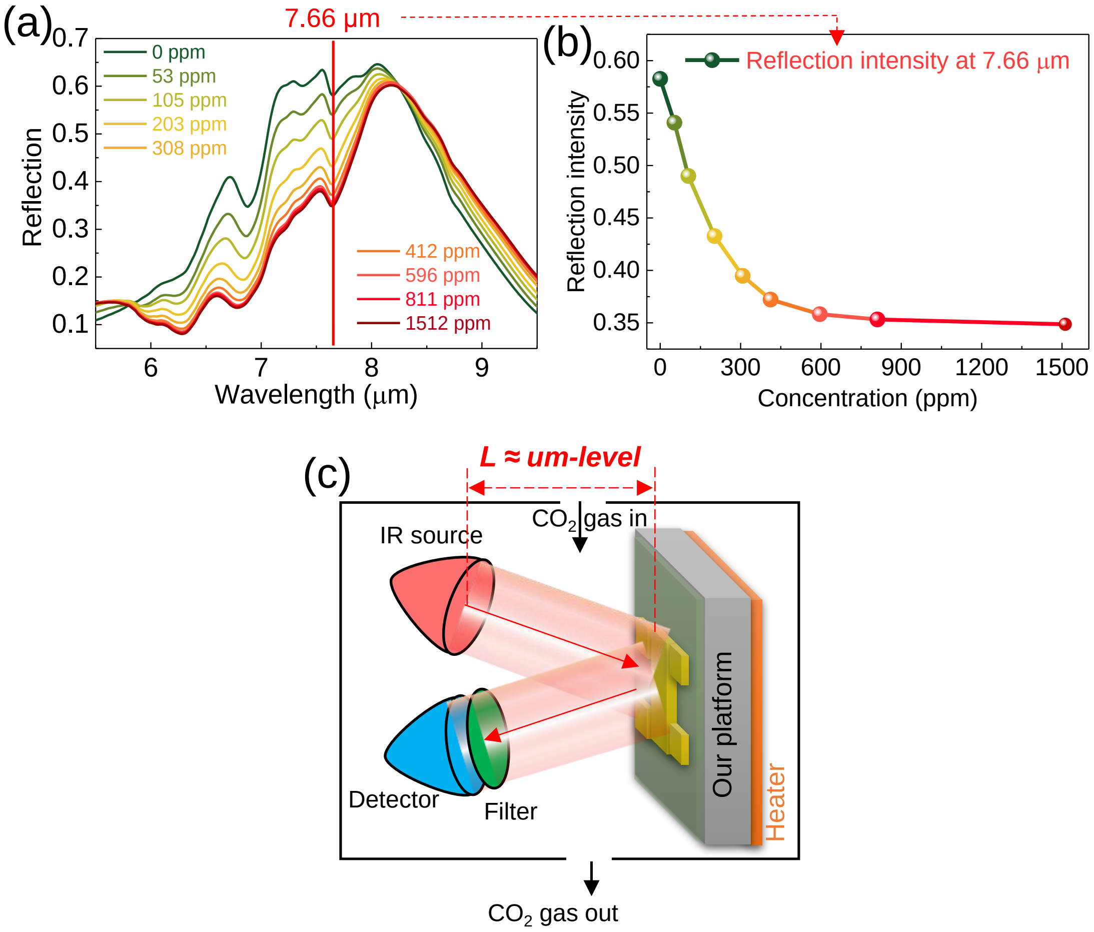

Fig. S15 Miniaturization of the platform. **a** Measurement results of our sensing platform at various CO_2 concentrations, which corresponds to Figure 5a of manuscript. **b** The reflection intensity at 7.66 μm as a function of CO_2 concentrations. **c** Conceptual diagram of the miniaturization of the platform

S16 Performance Comparison of Infrared CO_2 Sensors

Table S2 Comparison of the existing miniaturized CO_2 Gas sensors at mid-IR

Refs.	Method	Optical interaction length	Sensitivity	Limit of detection
Yasuda et al. [S35]	Commercial NDIR sensor;	140 mm	NA	60 ppm
Vincent et al. [S36]	MEMS miniaturization;	80 mm	NA	50 ppm
Li et al. [S37]	Graphene oxide enhancement; Microelectromechanical emitters;	~ 60 mm	NA	50 ppm
Mi et al. [S38]	Using silicon photonic refractometer;	20π μm	6×10^{-9} RIU/ppm	20 ppm
Starecki et al. [S39]	Fluorescence absorbance enhancement;	15 mm	NA	50 ppm
Ranacher et al. [S40]	Using infrared evanescent field absorption	5 mm	NA	< 500 ppm
Badri et al. [S41]	Using silicon subwavelength grating slot waveguide	14.11 μm	12.9 pm/ppm	210 ppm
Lochbaum et al. [S42]	Structure optimization; Using plasmonic thermal emitter;	4 mm	1.7×10^{-4} %/ppm	< 200 ppm
Lochbaum et al. [S43]	Structure optimization; Integrating optical cavity;	$5.7\times5.7\times4.5$ mm^3 cuboid	22.4±0.5 ppm/√Hz	< 200 ppm
Livingood et al. [S44]	Structure optimization; Using narrowband infrared emitting metamaterials	Centimeters	NA	50 ppm
Chen et al. [S45]	Structure optimization; Using a plasmonic thermal detector;	2.7 μm	3300 nm/RIU	100 ppm
Zhang et al. [S46]	Structure optimization; Using nanostructured covellite copper sulfide;	4 mm	NA	100 ppm

Refs.	Method	Optical interaction length	Sensitivity	Limit of detection
Chong et al. [S25]	Gas enrichment materials; Integrating plasmonic nanopatch metamaterial;	2.7 µm	NA	52 ppm
Kazanskiy et al. [S47]	Gas enrichment materials; Using silicon nano-cylinders design;	100 nm	17.3 pm/ppm	215 ppm
Bareza et al. [S48]	Gas enrichment materials; Graphene-based metamaterial;	30 nm	0.028 cm^{-1}/ppm	390 ppm
Zhou et al. [S20]	Gas enrichment materials; Dual-band absorber structure;	200 nm	0.0348‰/ppm	80 ppm
Hasan et al. [S49]	Gas enrichment materials; Using metamaterial absorber;	300 nm	0.0282%/ppm	40 ppm
Chang et al. [S24]	Gas enrichment materials; Using all-dielectric metamaterial	~155 nm	0.0537%/ppm	20 ppm
Wei et al. [S28]	Gas enrichment materials; Using crooked metamaterials;	150 nm	0.067%/ppm	30 ppm
**This work**	**MOF-PEI functionalized multi-hotspot strategy**	**180 nm**	**0.18%/ppm**	**ppb level**

S17 Comparison with Electrochemical and Photoacoustic CO_2 Sensors

There are three main types of mainstream technologies for the detection of CO_2 gas, namely electrochemical sensors, photoacoustic sensors, and infrared sensors. The introduction and comparison of these sensors are presented as follows.

Electrochemical CO_2 gas sensors measure the concentration of CO_2 gases by oxidizing or reducing the CO_2 gas at an electrode and measuring the resulting current. It consists of a sensing/working electrode, a solid-state electrolyte, and a reference electrode. An electrochemical CO_2 gas sensor has a simple structure of an electrochemical cell. In addition to the simple structure, its advantages also include linear output, low power consumption, and good resolution. However, since the state of the electrolyte is easily affected by the temperature and humidity of the working environment, chemical gas sensors are sensitive to temperature and humidity. Cross-sensitivity to other gases remains a challenge for electrochemical CO_2 gas sensors. Another advantage of the electrochemical CO_2 gas sensor is that it works well at high temperatures (up to 700 degrees Celsius) [S50].

Photoacoustic CO_2 gas sensors measure the concentration of CO_2 gases by using a microphone to detect vibrations and photoacoustic pressure waves that the gas generates after absorbing photon energy. It consists of a modulated light source to generate an intermittent light beam, and a sound transducer to detect the amplitude of the generated sound wave. In terms of acoustic

wave generation, acoustic resonators and quartz tuning forks could be used to enhance the intensity of generated acoustic waves [S51]. To increase the available optical power, optical resonators could be utilized. Similar to the infrared vibrations of molecules, an acoustic signal can only be generated when the emission spectrum of the light source and the absorption spectrum of the gas coincide. Therefore, it has good selectivity and low cross-sensitivity. In addition, it has the advantages of long-term stability, high sensitivity, etc. However, the performance of photoacoustic gas sensors depends on high-performance light sources and sound transducers, which makes it the most expensive sensor among these types of CO_2 gas sensors.

Nondispersive infrared (NDIR) CO_2 gas sensors are the most common infrared sensors for CO_2 gas detection [S52]. It is based on the absorption of infrared radiation at certain gas specific wavelengths in accordance with the Beer-Lambert law, which is expressed as $T=I/I_0=e^{-\varepsilon cL}$ (T is transmittance, I is the intensity of light passed through sample gas and an optical filter, I_0 is the initial light intensity emitted from the source, ε is the molar attenuation coefficient, c is gas concentration, and L is light path length). As observed, in order to achieve ppm level detection limit with a high signal-to-noise ratio, several centimeter length L is required. Therefore, the disadvantages of NDIR sensors include high detection limits and bulk dimensions. Its advantages are also significant, including long lifetime, high long-term stability, and low power consumption. Therefore, it is widely used in some harsh environments like mining projects, and its commercialization is the most successful of these sensors.

Our work is a kind of CO_2 gas sensor, and it utilizes the plasmonic enhancement effect of nanoantennas and the adsorption effect of MOF hybrids to achieve sensitive detection of CO_2 gas at a nanoscale light-mater interaction length. Besides, our detection limit reaches 1 ppm. Therefore, compared with NDIR sensors, our detection method has the following advantages: 1) shorter light-matter interaction length. For achieving ppm-level detection, the interaction length for infrared NDIR sensors is about 50 mm, and that of our method is 200 nm. Light-mater interaction length generally means the theoretical minimum size of the sensor. Although our current demonstration is bulky due to the use of the spectrometer's detector and light source, it can be improved by integrating the light source and detector with our chips, as discussed in the previous question; 2) Lower detection limit. The low detection limit allows our method to be suitable for some demanding application scenarios in industry and R&D. Notably, the nanoantenna-based gas sensing scheme in this manuscript does not conflict with the NDIR sensing technology, but is an improvement and supplement for the NDIR sensing technology, as demonstrated in previously reported works [S53]. The disadvantage of our method is that it is not as mature as NDIR technology, resulting in a low level of integration with light sources and detectors. Compared with electrochemical sensors, our detection method has the following advantages: 1) Lower detection limit. The reported detection limit of electrochemical CO_2 sensors is about tens of ppm [S54-S56]; 2) less sensitive to temperature and humidity; 3) less cross-sensitivity. Our method is based on the intrinsic infrared vibrational fingerprints of gas molecules; 4) longer lifetime. The advantage of electrochemical sensors includes simple structure, linear output, and stable operation at high temperatures. Compared with photoacoustic sensors, our detection method has the following advantages: 1) low cost. Our method does not require high-power light sources and an acoustic transducer; According to the reported reference [S52], the cost of photoacoustic sensors is about 100 times higher than infrared sensors. 2) low power consumption; Photoacoustic sensors require high energy to excite the acoustic waves from gas molecules. The advantage of photoacoustic sensors is long-term stability and high sensitivity. Overall, we demonstrate a CO_2 detection scheme with a low detection limit and high sensitivity and provide a promising idea for NDIR to reduce the light-matter interaction length.

Supplementary References

[S1] H. Aouani, H. Šípová, M. Rahmani, M. Navarrocia, K. Hegnerová, Ultrasensitive broadband probing of molecular vibrational modes with multifrequency optical antennas. ACS Nano **7**(1), 669–675 (2013). https://doi.org/10.1021/nn304860t

[S2] D. Yoo, D.A. Mohr, F. Vidal-Codina, A. John-Herpin, M. Jo et al., High-contrast infrared absorption spectroscopy via mass-produced coaxial zero-mode resonators with sub-10 nm gaps. Nano Lett. **18**, 1930–1936 (2018). https://doi.org/10.1021/acs.nanolett.7b05295

[S3] D. Ji, A. Cheney, N. Zhang, H. Song, J. Gao et al., Efficient mid-infrared light confinement within sub-5-nm gaps for extreme field enhancement. Adv. Opt. Mater. **5**(17), 1700223 (2017). https://doi.org/10.1002/adom.201700223

[S4] L. Dong, X. Yang, C. Zhang, B. Cerjan, L. Zhou et al., Nanogapped Au antennas for ultrasensitive surface-enhanced infrared absorption spectroscopy. Nano Lett. **17**(9), 5768-5774 (2017). https://doi.org/10.1021/acs.nanolett.7b02736

[S5] L.V. Brown, K. Zhao, N. King, H. Sobhani, P. Nordlander et al., Surface-enhanced infrared absorption using individual cross antennas tailored to chemical moieties. J. Am. Chem. Soc. **135**(9), 3688-3695 (2013). https://doi.org/10.1021/ja312694g

[S6] B. Metzger, M. Hentschel, T. Schumacher, M. Lippitz, X. Ye et al., Doubling the efficiency of third harmonic generation by positioning ITO nanocrystals into the hot-spot of plasmonic gap-antennas. Nano Lett. **14**(5), 2867-2872 (2014). https://doi.org/10.1021/nl500913t

[S7] L.V. Brown, X. Yang, K. Zhao, B.Y. Zheng, P. Nordlander et al., Fan-shaped gold nanoantennas above reflective substrates for surface-enhanced infrared absorption (SEIRA). Nano Lett. **15**(2), 1272-1280 (2015). https://doi.org/10.1021/nl504455s

[S8] W. Yue, V. Kravets, M. Pu, C. Wang, Z. Zhao et al., Multiple-resonant pad-rod nanoantennas for surface-enhanced infrared absorption spectroscopy. Nanotechnology **30**(46), 465206 (2019). https://doi.org/10.1088/1361-6528/ab3b69

[S9] R.T. Mackin, B. Cohn, B. Engelman, A. Goldner, I.V. Rubtsov et al., Plasmonic trimers for dual-frequency surface-enhanced two-dimensional infrared spectroscopy. J. Phys. Chem. C **123**(40), 24731-24739 (2019). https://doi.org/10.1021/acs.jpcc.9b07045

[S10] R. Semenyshyn, M. Hentschel, C. Huck, J. Vogt, F. Weiher et al., Resonant plasmonic nanoslits enable in vitro observation of single-monolayer collagen-peptide dynamics. ACS Sens. **4**(8), 1966-1972 (2019). https://doi.org/10.1021/acssensors.9b00377

[S11] C. Chen, D.A. Mohr, H.K. Choi, D. Yoo, M. Li et al., Waveguide-integrated compact plasmonic resonators for on-chip mid-infrared laser spectroscopy. Nano Lett. **18**(12), 7601-7608 (2018). https://doi.org/10.1021/acs.nanolett.8b03156

[S12] A. John-Herpin, A. Tittl, H. Altug, Quantifying the limits of detection of surface-enhanced infrared spectroscopy with grating order-coupled nanogap antennas. ACS Photonics **5**(10), 4117-4124 (2018). https://doi.org/10.1021/acsphotonics.8b00847

[S13] Y. Chen, S. Zhang, Z. Shu, Z. Wang, P. Liu et al., Adhesion-engineering-enabled “sketch and peel” lithography for aluminum plasmonic nanogaps. Adv. Opt. Mater. **8**(2), 1901202 (2019). https://doi.org/10.1002/adom.201901202

[S14] I. Hwang, M. Kim, J. Yu, J. Lee, J.H. Choi et al., Ultrasensitive molecule detection based on infrared metamaterial absorber with vertical nanogap. Small Methods **5**(8), 2100277 (2021). https://doi.org/10.1002/smtd.202100277

[S15] I. Hwang, J. Yu, J. Lee, J.H. Choi, D.G. Choi et al., Plasmon-enhanced infrared spectroscopy based on metamaterial absorbers with dielectric nanopedestals. ACS

Photonics **5**(9), 3492-3498 (2018). https://doi.org/10.1021/acsphotonics.8b00702

[S16] J.Y. Jung, J. Lee, J.H. Choi, D.G. Choi, J.H. Jeong, Enhancement of refractive index sensing for an infrared plasmonic metamaterial absorber with a nanogap. Opt. Express **29**(14), 22796-22804 (2021). https://doi.org/10.1364/OE.432392

[S17] D.S. Su, D.P. Tsai, T.J. Yen, T. Tanaka, Ultrasensitive and selective gas sensor based on a channel plasmonic structure with an enormous hot spot region. ACS Sens. **4**(11), 2900-2907 (2019). https://doi.org/10.1021/acssensors.9b01225

[S18] H. Zhou, D.X. Li, X.D. Hui, D.L. Hu, X. Chen et al., Metamaterial gas sensing platform based on surface-enhanced infrared absorption. 2020 33rd IEEE Int. Conf. Micro Electro Mechan. Syst., Vancouver, BC, Canada, 717-720 (January, 2020). https://doi.org/10.1109/MEMS46641.2020.9056404

[S19] T.H.H. Le, T. Tanaka, Plasmonics-nanofluidics hydrid metamaterial: an ultrasensitive platform for infrared absorption spectroscopy and quantitative measurement of molecules. ACS Nano **11**(10), 9780-9788 (2017). https://doi.org/10.1021/acsnano.7b02743

[S20] H. Zhou, X. Hui, D. Li, D. Hu, X. Chen et al., Metal-organic framework-surface-enhanced infrared absorption platform enables simultaneous on-chip sensing of greenhouse gases. Adv. Sci. **7**(20), 2001173 (2020). https://doi.org/10.1002/advs.202001173

[S21] H. Zhou, D. Li, X. Hui, X. He, H. Huang et al., Mid-IR metamaterial absorber with polyvinylamine as a sensitive layer for on-chip sensing of carbon dioxide. 2021 21st Int. Conf. Solid-State Sens. Actuat. Microsyst., Orlando, FL, USA, 859-862 (June, 2021). https://doi.org/10.1109/Transducers50396.2021.9495629

[S22] A.E. Cetin, D. Etezadi, H. Altug, Accessible nearfields by nanoantennas on nanopedestals for ultrasensitive vibrational spectroscopy. Adv. Opt. Mater. **2**(9), 866-872 (2014). https://doi.org/10.1002/adom.201400171

[S23] T.H.H. Le, A. Morita, K. Mawatari, T. Kitamori, T. Tanaka, Metamaterials-enhanced infrared spectroscopic study of nanoconfined molecules by plasmonics-nanofluidics hydrid device. ACS Photonics **5**(8), 3179-3188 (2018). https://doi.org/10.1021/acsphotonics.8b00398

[S24] Y. Chang, D. Hasan, B. Dong, J. Wei, Y. Ma et al., All-dielectric surface-enhanced infrared absorption-based gas sensor using guided resonance. ACS Appl. Mater. Interfaces **10**(44), 38272-38279 (2018). https://doi.org/10.1021/acsami.8b16623

[S25] X. Chong, Y. Zhang, E. Li, K.J. Kim, P.R. Ohodnicki et al., Surface-enhanced infrared absorption: pushing the frontier for on-chip gas sensing. ACS Sens. **3**(1), 230-238 (2018). https://doi.org/10.1021/acssensors.7b00891

[S26] N. Bareza, B. Paulillo, T.M. Slipchenko, M. Autore, I. Dolado et al., Phonon-enhanced mid-infrared CO_2 gas sensing using boron nitride nanoresonators. ACS Photonics **9**(1), 34-42 (2022). https://doi.org/10.1021/acsphotonics.1c01254

[S27] J. Xu, Z. Ren, B. Dong, X. Liu, C. Wang et al., Nanometer-scale heterogeneous interfacial sapphire wafer-bonding for enabling plasmonic-enhanced nanofluidic mid-infrared spectroscopy. ACS Nano **14**, 12159−12172 (2020). https://doi.org/10.1021/acsnano.0c05794

[S28] J. Wei, Y. Li, Y. Chang, D.M.N. Hasan, B. Dong et al., Ultrasensitive transmissive infrared spectroscopy via loss engineering of metallic nanoantennas for compact devices. ACS Appl. Mater. Interfaces **11**(50), 47270–47278 (2019). https://doi.org/10.1021/acsami.9b18002

[S29] Z. Ren, Z. Zhang, J. Wei, H. Wang, B. Dong et al., Machine learning augmented VOC identification by mid-infrared nanoantennas with microfluidics chambers. 2021 21st

Int. Conf. Solid-State Sens. Actuat. Microsyst., Orlando, FL, USA, 389-392 (June, 2021). https://doi.org/10.1109/Transducers50396.2021.9495761

[S30] R. Adato, A. Artar, S. Erramilli, H. Altug, Engineered absorption enhancement and induced transparency in coupled molecular and plasmonic resonator systems. Nano Lett. **13**(6), 2584-2591 (2013). https://doi.org/10.1021/nl400689q

[S31] J. Wei, D. Hasan, C. Lee, An optimized nanoantenna platform for surface enhanced mid-infrared sensing. 2019 20th Int. Conf. Solid-State Sens. Actuat. Microsyst. Eurosens. XXXIII, Berlin, Germany, 1157-1160 (June, 2019). https://doi.org/10.1109/TRANSDUCERS.2019.8808771

[S32] S. Yuan, D. Naveh, K. Watanabe, T. Taniguchi, F. Xia, A wavelength-scale black phosphorus spectrometer. Nat. Photonics **15**(8), 601-607 (2021). https://doi.org/10.1038/s41566-021-00787-x

[S33] V.R. Shrestha, B. Craig, J. Meng, J. Bullock, A. Javey et al., Mid- to long-wave infrared computational spectroscopy with a graphene metasurface modulator. Sci. Rep. **10**(1), 5377 (2020). https://doi.org/10.1038/s41598-020-61998-w

[S34] A. Tittl, A. Leitis, M. Liu, F. Yesilkoy, D.Y. Choi et al., Imaging-based molecular barcoding with pixelated dielectric metasurfaces. Science **360**(6393), 1105-1109 (2018). https://doi.org/10.1126/science.aas9768

[S35] T. Yasuda, S. Yonemura, A. Tani, Comparison of the characteristics of small commercial NDIR CO_2 sensor models and development of a portable CO_2 measurement device. Sensors **12**(3), 3641-3655 (2012). https://doi.org/10.3390/s120303641

[S36] T.A. Vincent, J.W. Gardner, A low cost mems based NDIR system for the monitoring of carbon dioxide in breath analysis at ppm levels. Sens. Actuators B **236**, 954-964 (2016). https://doi.org/10.1016/j.snb.2016.04.016

[S37] N. Li, H. Yuan, L. Xu, J. Tao, D.K.T. Ng et al., Radiation enhancement by graphene oxide on microelectromechanical system emitters for highly selective gas sensing. ACS Sens. **4**(10), 2746-2753 (2019). https://doi.org/10.1021/acssensors.9b01275

[S38] G. Mi, C. Horvath, M. Aktary, V. Van, Silicon microring refractometric sensor for atmospheric CO_2 gas monitoring. Opt. Express **24**(2), 1773-1780 (2016). https://doi.org/10.1364/OE.24.001773

[S39] F. Starecki, F. Charpentier, J.L. Doualan, L. Quetel, K. Michel et al., Mid-IR optical sensor for CO_2 detection based on fluorescence absorbance of DY^{3+}:$Ga_5Ge_{20}Sb_{10}S_{65}$ fibers. Sens. Actuators B **207**, 518-525 (2015). https://doi.org/10.1016/j.snb.2014.10.011

[S40] C. Ranacher, C. Consani, N. Vollert, A. Tortschanoff, M. Bergmeister et al., Characterization of evanescent field gas sensor structures based on silicon photonics. IEEE Photonics J. **10**(5), 1-14 (2018). https://doi.org/10.1109/jphot.2018.2866628

[S41] S.H. Badri, Transmission resonances in silicon subwavelength grating slot waveguide with functional host material for sensing applications. Opt. Laser Technol. **136**, 106776 (2021). https://doi.org/10.1016/j.optlastec.2020.106776

[S42] A. Lochbaum, Y. Fedoryshyn, A. Dorodnyy, U. Koch, C. Hafner et al., On-chip narrowband thermal emitter for mid-IR optical gas sensing. ACS Photonics **4**(6), 1371-1380 (2017). https://doi.org/10.1021/acsphotonics.6b01025

[S43] A. Lochbaum, A. Dorodnyy, U. Koch, S.M. Koepfli, S. Volk et al., Compact mid-infrared gas sensing enabled by an all-metamaterial design. Nano Lett. **20**(6), 4169-4176 (2020). https://doi.org/10.1021/acs.nanolett.0c00483

[S44] A. Livingood, J.R. Nolen, T.G. Folland, L. Potechin, G. Lu et al., Filterless nondispersive infrared sensing using narrowband infrared emitting metamaterials.

ACS Photonics **8**(2), 472-480 (2021). https://doi.org/10.1021/acsphotonics.0c01432

[S45] Q. Chen, L. Liang, Q. Zheng, Y. Zhang, L. Wen, On-chip readout plasmonic mid-IR gas sensor. Opto-Electron. Adv. **3**(7), 190040-190040 (2020). https://doi.org/10.29026/oea.2020.190040

[S46] Y. Zhang, X. Chong, H. Sun, M.M. Kedir, K.J. Kim et al., Nanostructured copper sulfide thin film via a spatial successive ionic layer adsorption and reaction process showing significant surface-enhanced infrared absorption of CO_2. J. Mater. Chem. C **8**(9), 3069-3078 (2020). https://doi.org/10.1039/c9tc06423k

[S47] N.L. Kazanskiy, M.A. Butt, S.N. Khonina, Carbon dioxide gas sensor based on polyhexamethylene biguanide polymer deposited on silicon nano-cylinders metasurface. Sensors **21**(2), 378 (2021). https://doi.org/10.3390/s21020378

[S48] N. Bareza, K.K. Gopalan, R. Alani, B. Paulillo, V. Pruneri, Mid-infrared gas sensing using graphene plasmons tuned by reversible chemical doping. ACS Photonics **7**(4), 879-884 (2020). https://doi.org/10.1021/acsphotonics.9b01714

[S49] D. Hasan, C. Lee, Hybrid metamaterial absorber platform for sensing of CO_2 gas at mid-IR. Adv. Sci. **5**(5), 1700581 (2018). https://doi.org/10.1002/advs.201700581

[S50] M. Alonso-Porta, R. Kumar, Use of NASICON/Na_2Co_3 system for measuring CO_2. Sens. Actuators B **71**, 173-178 (2000). https://doi.org/10.1016/S0925-4005(00)00600-6

[S51] S. Palzer, Photoacoustic-based gas sensing: a review. Sensors **20**(9), 2745 (2020). https://doi.org/10.3390/s20092745

[S52] L.B. Mendes, N.W. Ogink, N. Edouard, H.J. Dooren, F. Tinoco Ide et al., NDIR gas sensor for spatial monitoring of carbon dioxide concentrations in naturally ventilated livestock buildings. Sensors **15**(5), 11239-11257 (2015). https://doi.org/10.3390/s150511239

[S53] Y. Nishijima, Y. Adachi, L. Rosa, S. Juodkazis, Augmented sensitivity of an IR-absorption gas sensor employing a metal hole array. Opt. Mater. Express **3**(7), 968-976 (2013). https://doi.org/10.1364/ome.3.000968

[S54] N.J. Choi, H.K. Lee, S.E. Moon, W.S. Yang, J. Kim, Stacked-type potentiometric solid-state CO_2 gas sensor. Sens. Actuators B Chem. **187**, 340-346 (2013). https://doi.org/10.1016/j.snb.2012.12.037

[5S5] J.W. Fergus, A review of electrolyte and electrode materials for high temperature electrochemical CO_2 and SO_2 gas sensors. Sens. Actuators B **134**(2), 1034-1041 (2008). https://doi.org/10.1016/j.snb.2008.07.005

[S56] T.W. Kim, J.W. Kim, S.M. Lee, C.O. Park, Humidity effects on the initial stabilization behavior of a solid electrochemical CO_2 sensor. Sens. Actuators B **295**, 65-69 (2019). https://doi.org/10.1016/j.snb.2019.05.015
